# Human Lactate Dehydrogenase A Inhibitors: A Molecular Dynamics Investigation

**DOI:** 10.1371/journal.pone.0086365

**Published:** 2014-01-17

**Authors:** Yun Shi, B. Mario Pinto

**Affiliations:** Department of Chemistry, Simon Fraser University, Burnaby, British Columbia, Canada; Instituto de Tecnologica Química e Biológica, UNL, Portugal

## Abstract

Lactate dehydrogenase A (LDHA) is an important enzyme in fermentative glycolysis, generating most energy for cancer cells that rely on anaerobic respiration even under normal oxygen concentrations. This renders LDHA a promising molecular target for the treatment of various cancers. Several efforts have been made recently to develop LDHA inhibitors with nanomolar inhibition and cellular activity, some of which have been studied in complex with the enzyme by X-ray crystallography. In this work, we present a molecular dynamics (MD) study of the binding interactions of selected ligands with human LDHA. Conventional MD simulations demonstrate different binding dynamics of inhibitors with similar binding affinities, whereas steered MD simulations yield discrimination of selected LDHA inhibitors with qualitative correlation between the *in silico* unbinding difficulty and the experimental binding strength. Further, our results have been used to clarify ambiguities in the binding modes of two well-known LDHA inhibitors.

## Introduction

An emerging hallmark of cancer is its altered cell energy metabolism that favors anaerobic respiration over aerobic respiration. [1], [2] Unlike normal cells that utilize the Krebs cycle as the major energy-producing process in the presence of adequate oxygen, many cancer cells preferentially derive ATP through glycolysis, followed by fermentation that converts pyruvate to lactate. The preference towards fermentative glycolysis (anaerobic respiration), regardless of oxygen availability in the environment, is known as the Warburg effect. [3] This effect confers a significant growth advantage for cancer cells within a hypoxic environment, [4] and thus new cancer therapies can be developed by targeting the processes of glycolysis and fermentation used by cancer cells.

Lactate dehydrogenase (LDH) is an enzyme that catalyzes the interconversion of pyruvate-NADH and lactate-NAD^+^, critical for anaerobic respiration as it can recycle NAD^+^ for the continuation of glycolysis. [5], [6] Two major isoforms of LDH, namely LDHA (LDHM or LDH5) and LDHB (LDHH or LDH1), exist in mammalian cells, with the A form favoring the transformation of pyruvate to lactate and the B form favoring the backward conversion. [7] Hence, human LDHA could be a molecular target for the inhibition of fermentative glycolysis and thus the growth and proliferation of cancer cells. Indeed, it is required for the initiation, maintenance, and progression of tumors. [8], [9] In addition, up-regulation of LDHA is characteristic of many cancer types, [10], [11], [12], [13], [14] and inhibition of LDHA by small molecules has been found to confer antiproliferative activity. [9], [15] More importantly, complete deficiency of LDHA does not give rise to any symptoms in humans under normal circumstances, [16] indicating that selective LDHA inhibitors should only present minimal side effects. Therefore, LDHA is considered an attractive molecular target for the development of novel anticancer agents.

**Figure 1:**
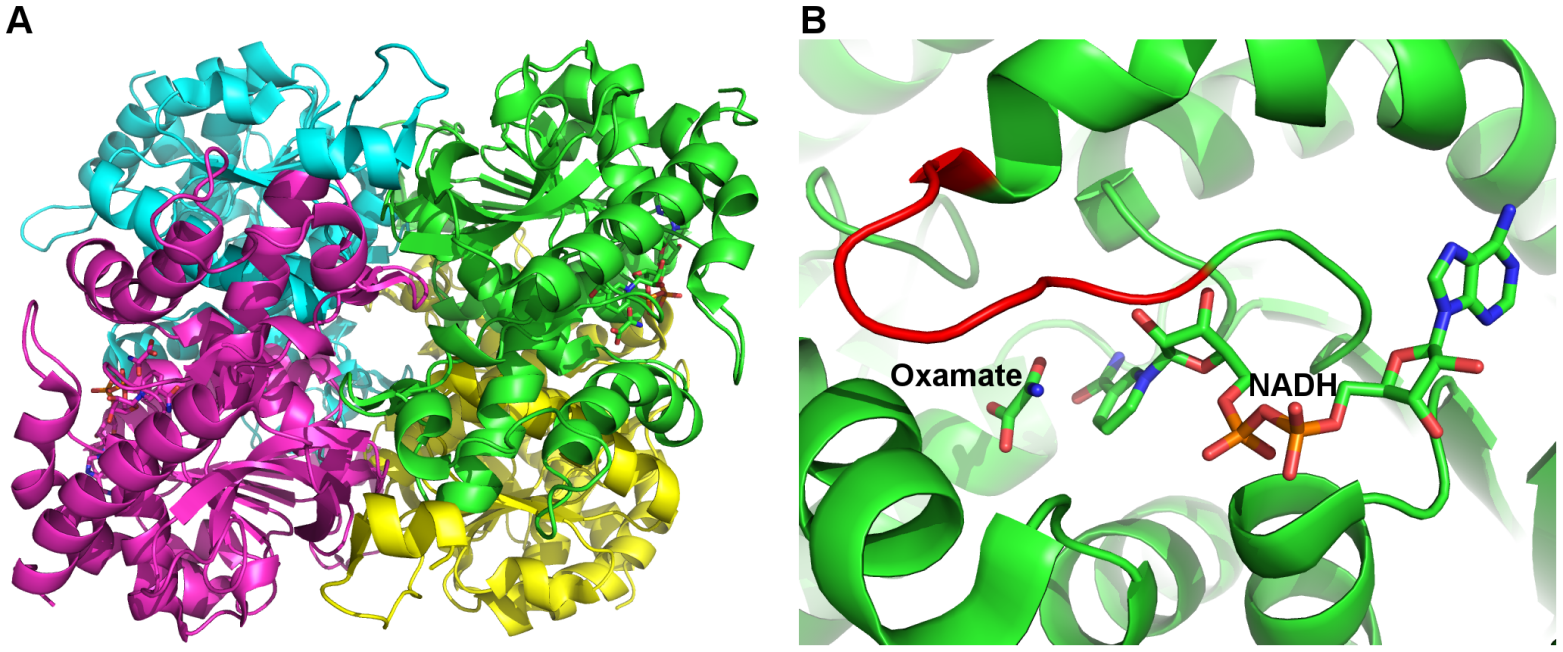
Structure of human LDHA (PDB 1I10). Amino acid residues are shown in cartoons and NADH/oxamate are shown in sticks. A) Tetrameric structure of human LDHA. Chains A, B, C, and D are colored green, yellow, magenta, and cyan, respectively. B) Close-up view of the binding site from chain A. The active site mobile loop is colored red.

**Figure 2:**
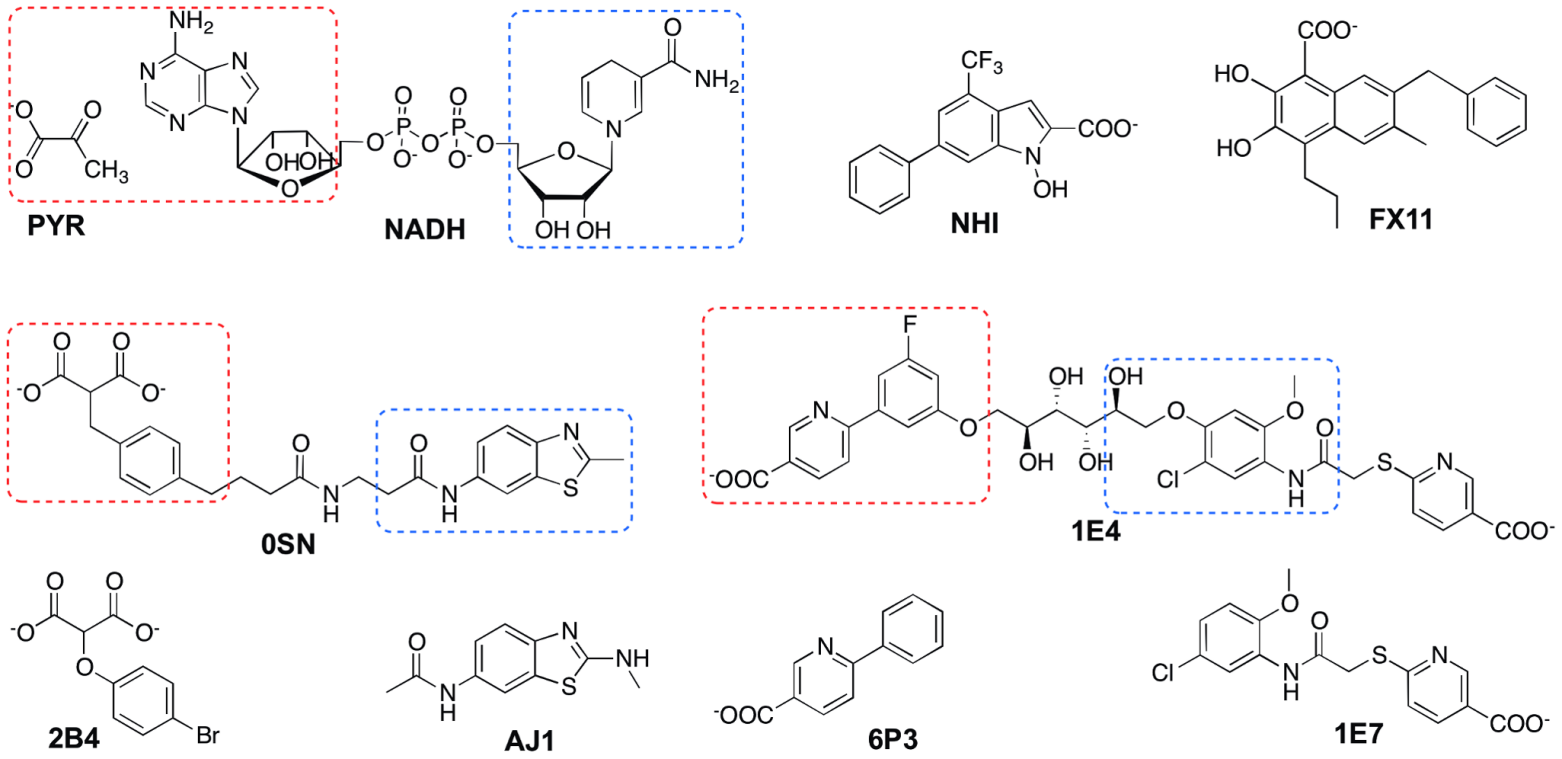
Structures of LDHA binders of interest. A-site and S-site binding moieties are indicated by boxes with blue dashed lines and red dashed lines, respectively.

Human LDHA has a tetrameric structure with four identical monomers, each in possession of its own NADH cofactor binding site and substrate binding site (Figure 1A). [17] The cofactor binds to LDHA in an extended conformation, with its nicotinamide group forming part of the substrate binding site (Figure 1B). [17] The closure of a mobile loop (residues 96–107; residue numbering refers to human LDHA in PDB 1I10), in which the conserved Arg105 could stabilize the transition state in the hydride-transfer reaction, is indispensible for catalytic activity. [17] Yet, the first human LDHA structure (PDB 1I10), in complex with a substrate mimic (oxamate) and the cofactor NADH, shows that the mobile loop of one of the four identical monomers, chain D, is in an open conformation, indicating certain probability of the loop being open. There have been several efforts to develop human LDHA inhibitors, [15], [18], [19], [20], [21] and crystal structures are available for complexes of some inhibitors and LDHAs from human, rat, and rabbit. [18], [19], [20], [21] A fragment-based approach has been successfully employed to combine adenosine-site (A-site) binders and nicotinamide/substrate-site (S-site) binders, yielding dual-site binders with nanomolar binding affinities (Figure 2 and Table 1). [18], [19].

**Table 1:** Binding constants and site of binding of LDHA binders.

Ligand	*K*_d_ (µM) [15], [18], [19], [22], [53]	Site of binding [15], [18], [19], [22], [53]
PYR^a^	3.9	S
NADH	0.62	dual
0SN	0.093	dual
2B4	210	S
AJ1	770	A
1E4	0.068	dual
6P3	2300	S
1E7	137	A
NHI	9	unknown
FX11	0.05	unknown

^a^ The natural substrate pyruvate is referred to as PYR hereafter for simplicity.

However, the binding dynamics of these LDHA binders have not been thoroughly studied. In addition, the binding location and geometry of two important inhibitors, NHI and FX11 (Table 1), proven to be NADH-competitive and have antiproliferative activities against cancer cell lines, [9], [15], [22] are not clear. The *in silico* discrimination of inhibitors in terms of binding strengths is also desirable. Therefore, we present a computational approach herein to examine the binding of a variety of human LDHA inhibitors (Figure 2) to complement previous experimental studies. This approach includes both conventional and steered molecular dynamics (MD) simulations with sufficient system size to probe the dynamics and strength of inhibitor binding.

## Results

Conventional MD simulations were performed in triplicate for each system of human LDHA:ligand complex for 60 ns. Root mean squared deviations of both LDHA backbone atoms and binding site heavy atoms stabilized within 40 ns for most trajectories (Texts S2 and S3), indicating good convergence of our MD simulations. As a result, all analyses were conducted on the final 20 ns (40–60 ns) of each MD trajectory, unless otherwise indicated. For LDHA:PYR-NADH, LDHA:2B4, and LDHA:NHI_S_, only chains A, B, and C were analyzed since these systems were shown to prefer a loop-closed conformation (Table 2) while the loop in chain D stayed open (see below). For other LDHA:ligand systems, all four monomers from triplicate runs were subjected to analysis.

**Table 2:** Populations of different loop conformations.

		Loop conformations^a^		
System^b^		Closed	Intermediate	Open
	LDHA apo	50%	11%	39%
Dual-sitebinding	LDHA:PYR-NADH	49%	7%	44%
	LDHA:0SN	89%	10%	1%
	LDHA:1E4	18%	40%	42%
A-sitebinding	LDHA:AJ1	51%	14%	35%
	LDHA:1E7	52%	16%	32%
	LDHA:NHI_A_	12%	16%	72%
	LDHA:FX11_A_	48%	16%	36%
S-sitebinding	LDHA:2B4	96%	4%	0
	LDHA:6P3	41%	18%	41%
	LDHA:NHI_S_	69%	6%	25%
	LDHA:FX11_S_	13%	7%	80%

^a^ The center-of-mass distance between alpha carbons of four loop residues (100–103) and those of Tyr238 and Lys242, which reside on the opposite side of the binding groove, was used to define loop conformations: distance smaller than 0.9 nm, closed; larger than 1.05 nm, open; between 0.9 nm and 1.05 nm, intermediate.

^b^ Only monomers with the mobile loop initially closed are considered.

### Monomer vs Tetramer

**Figure 3:**
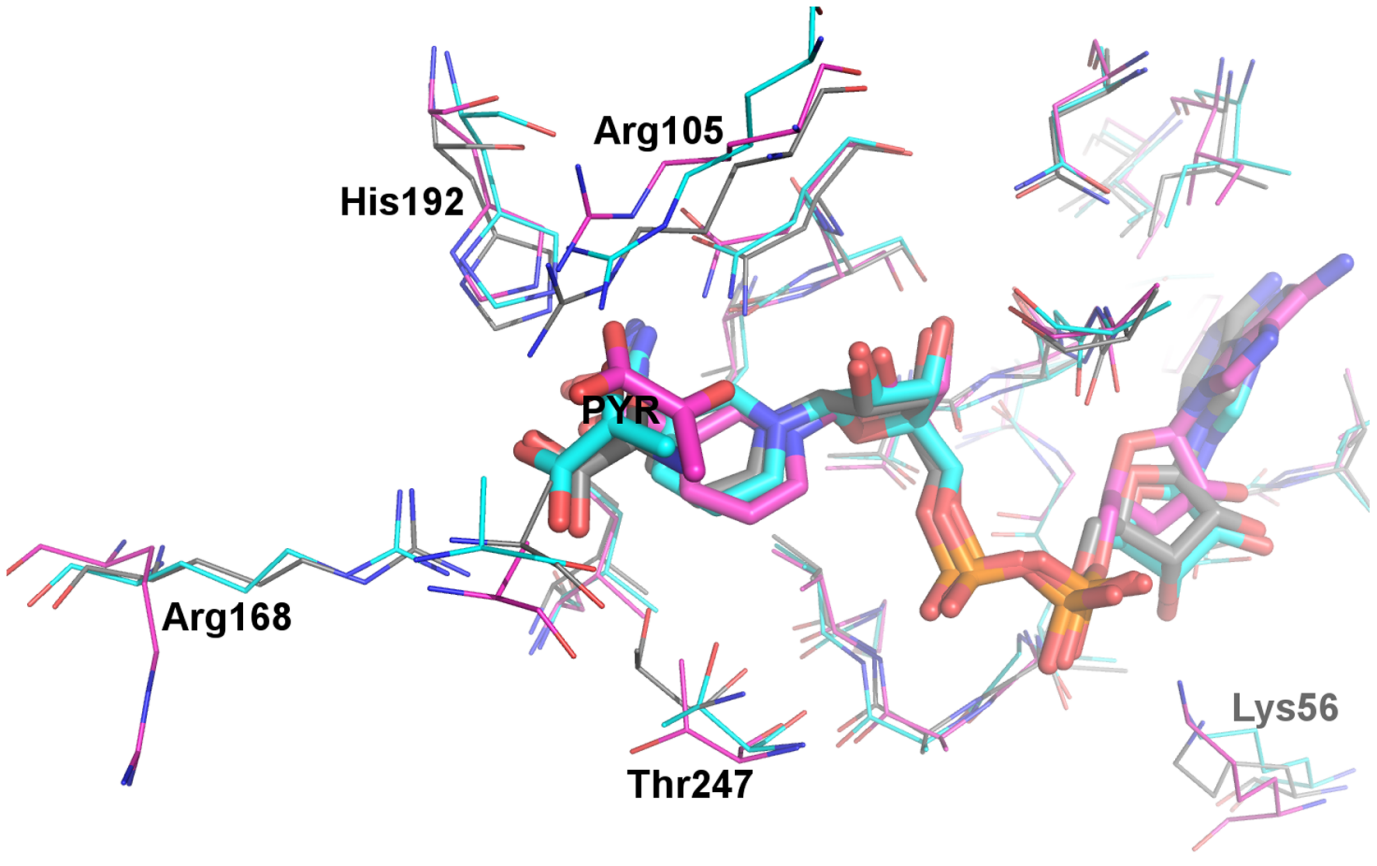
Comparison of monomeric and tetrameric MD models of LDHA:PYR-NADH. Representative structures of the monomeric (carbon atoms in magenta) and the tetrameric (carbon atoms in cyan) forms are overlaid with the crystal structure (PDB 1I10, carbon atoms in grey). Selected binding site residues are shown in thin lines, while pyruvate and NADH are shown in thick sticks. Other atoms are colored: oxygen, red; nitrogen, blue; phosphate, orange.

It is tempting to model a monomeric enzyme-ligand complex or even a truncated enzyme-ligand complex in MD simulations, as this saves significant computational costs and may still generate reasonable enzyme-ligand interaction patterns. To assess the validity of such an approach, the native system LDHA:PYR-NADH was modeled in both the monomeric and tetrameric forms. During the MD simulations of the monomeric LDHA:PYR-NADH system, the N-terminal arm (residues 1–20), which interacts with the other two subunits in tetrameric LDHA, wrapped towards the main body (residues 21–331). While some binding site residues showed slightly different orientations and positions, key interactions of substrate binding were significantly different between monomeric and tetrameric forms (Figure 3). The substrate in the monomeric form was unable to establish simultaneous contacts with Arg105 and Arg168 (Table 3), both of which were known to provide important polar interactions for substrate binding (Figure 3). Additionally, three hydrogen bonds donated to the substrate were mostly lost/replaced in the monomeric form (Table 3). In tetrameric LDHA, however, the substrate was relatively static, engaging in simultaneous contacts with both arginines and preserving the three hydrogen bonds donated to the substrate during most of the simulation (Table 3). The NADH binding did not show significant differences between monomeric and tetrameric forms within the simulation timescale. In light of the improper substrate binding patterns from modeling of monomeric LDHA:PYR-NADH, MD simulations of all other systems were performed in the tetrameric form.

**Table 3:** Populations of important substrate binding interactions in simulations of monomeric vs tetrameric forms.

Contacts^a^ with Arg105 and Arg168	Both	Either	None
Monomeric form	0	99%	1%
Tetrameric form	77%	17%	6%
**Hydrogen bonding interaction**	Asn137 ND2 and PYR O^b^	His192 NE2 and PYR O^b^	Thr247 OG1 and PYR OX2^b^
Monomeric form	8%	16%	0
Tetrameric form	69%	70%	68%

^a^ Contacts are defined as any heavy atom pair with distance ≤0.4 nm.

^b^ O refers to the carbonyl oxygen of PYR, while OX2 refers to a carboxylate oxygen (Text S1).

### The Mobile Loop

While the initial structures of LDHA:0SN and LDHA:1E4 systems were built with all mobile loops closed (from PDB 4AJP), all other LDHA:ligand systems had the mobile loop of chain D initially open (from PDB 1I10). Throughout our conventional MD simulations of all LDHA:ligand systems, no complete and stable closure of the mobile loop on chain D was observed, although transient loop closure lasting less than 1 ns was observed for apo LDHA and LDHA:FX11_A_ systems. The loop closure is believed to be the rate-limiting step in the turnover of LDHA. [23] In a pig LDHA:oxamate-NADH system, this process takes more than 1 ms to occur, [24] a time scale currently inaccessible to regular all-atom, explicit solvent MD simulations of such a large system. Nevertheless, opening of the mobile loop that was initially in the closed conformation occurred in all systems to different extents (Table 2). This suggests that loop opening occurs within a shorter time scale and the open conformation is probably energetically favorable in the absence of strong interactions between the ligand and mobile loop residues. Of note, the closure of the mobile loop is not necessarily required for ligand binding within the S-site, and certain S-site binders may force the loop open when they bind. [20].

### Dual-site Binders

**Figure 4:**
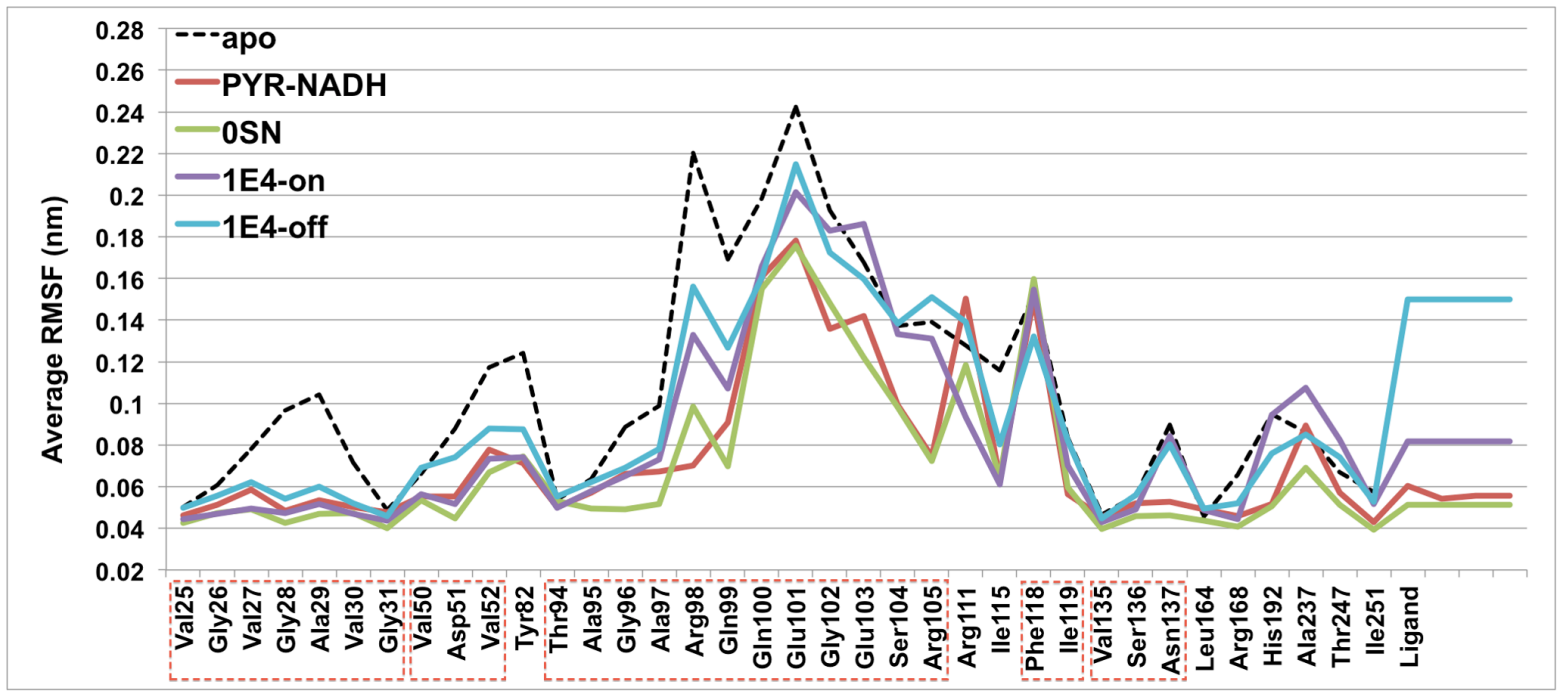
Root-mean-squared fluctuations (RMSF) of dual-site binding systems. “1E4-on” represents data from trajectories where 1E4 had strong ionic interactions with Arg111, while “1E4-off” indicates data from trajectories without such interactions. Contiguous residues are labeled by boxes with red dashed lines.

The two dual-site binders selected for MD simulations have similar binding affinities, but the binding site dynamics of LDHA:1E4 and LDHA:0SN complexes turned out to be different (Figure 4, Tables 4 and S2). The nicotinate moiety on the A-site end (A-end) of 1E4 extends outside the binding groove, [19] and was constantly fluctuating during MD simulations when its carboxylate group was not involved in strong ionic interactions with Arg111 above the binding groove. This could be caused by the pointing away of the Arg111 side chain from the nicotinate in the initial structure, since trajectories with the establishment of such ionic interactions showed much smaller fluctuations of binding site residues than those without such interactions (Figure 4). The absence of this interaction also resulted in notably different bound conformations for 1E4 (Text S4).

**Table 4:** Percentage existence of ionic interactions between LDHA and ligands.

		Positively charged group (guanidinium or imidazolium)				
		Arg98	Arg105	Arg111	Arg168	His192
Negatively charged group (phosphate or carboxylate)	NADH α^a^	70.1				
	NADH β^b^	97.3				
	PYR		39.4		94.1	88.1
	0SN α^c^		96.4			98.0
	0SN β^d^		78.5		100	65.2
	1E4 α^e^			30.5		
	1E4 β^f^				90.0	69.5
	1E7			19.3		
	NHI_A_	22.6				
	FX11_A_			21.8		
	2B4 α^c^		99.9			97.3
	2B4 β^d^		69.9		100	37.7
	6P3		23.0		74.9	73.3
	NHI_S_		79.4		13.6	91.5
	FX11_S_				53.5	58.3

^a^ The phosphate group linked to adenosine.

^b^ The phosphate group linked to nicotinamide riboside.

^c^ The carboxylate group adjacent to Arg105 (mobile loop).

^d^ The carboxylate group adjacent to Arg168.

^e^ The carboxylate group within A-site.

^f^ The carboxylate group within S-site.

Analogous to the adenine ring of NADH, the benzothiazole ring of 0SN and the 2-chloro-5-methoxyphenyl ring of 1E4 were stabilized by hydrophobic interactions with Val52, Ala95, and Ile115 within the A-site. Whereas the two hydroxyl groups on the adenosine ribose ring donated hydrogen bonds to Asp51, the amide nitrogen attached to the benzothiazole ring of 0SN also formed a hydrogen bond with Asp51 during most of the trajectory (Table S2). Yet, the hydroxyl groups on 1E4 were unable to engage in direct hydrogen bonding interaction with Asp51 for most of the time, probably due to their exposure to the bulk solvent and the formation of water-mediated hydrogen bonds. [19] Another hydrogen bond in the crystal structure of rabbit LDHA:1E4, between a hydroxyl group and Thr94 O, was also mostly absent during our MD simulations. Nevertheless, Gly96 N donated a hydrogen bond to 1E4, mimicking the two hydrogen bonds between Gly96 and 0SN, which were well maintained throughout the simulation of LDHA:0SN system (Table S2).

Notably, Arg98 exhibited larger fluctuations in both LDHA:0SN and LDHA:1E4 systems than in the native LDHA:PYR-NADH system (Figure 4). Apparently, no chemical moieties in either 0SN or 1E4 could mimic the negatively charged diphosphate group of NADH (Table 4), and thus neither 0SN nor 1E4 were able to hold the Arg98 side chain in a relatively static orientation.

**Figure 5:**
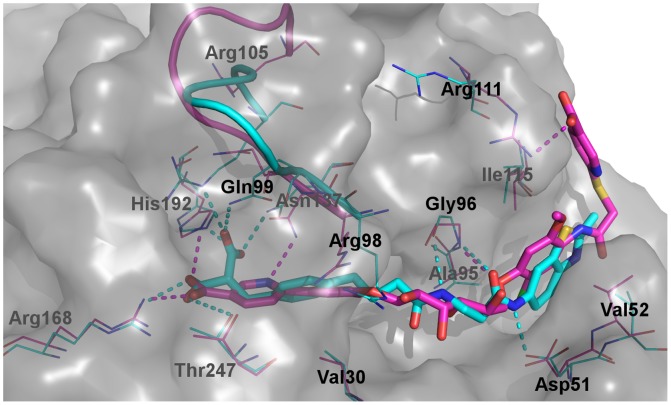
Comparison of the binding of dual-site inhibitors. Representative MD snapshots of LDHA:0SN (carbon atoms in cyan) and LDHA:1E4 (carbon atoms in magenta) are superimposed. Selected binding site residues are labeled and shown in thin lines, while 0SN and 1E4 are shown in thick sticks. The mobile loop is represented by a ribbon, and the solvent-accessible surface of LDHA is indicated by a grey transparent surface. Dashed lines represent polar interactions. Other atoms are colored: oxygen, red; nitrogen, blue; sulfur, yellow; chlorine, green; fluorine, pale cyan.

Within the S-site, hydrophobic interactions with Val30 were well maintained in both LDHA:0SN and LDHA:1E4 by their phenyl rings. In addition to hydrogen bonding interactions with Asn137 ND2 and Thr247 OG1, 0SN also accepted a hydrogen bond from Gln99 NE2 (Figure 5 and Table S2). In LDHA:1E4, however, these hydrogen bonds existed less frequently (Table S2). Interestingly, the pyridine ring within the S-site rotated almost 180 degrees during some of the MD simulations, leading to the formation of a hydrogen bond between the pyridine ring nitrogen and Asn137 ND2 (Figure 5).

Unlike the di-carboxylate of 0SN that maintained strong ionic interactions with Arg105, Arg168, and His192 throughout the simulation, the nicotinate of 1E4 within the S-site was not able to establish strong interactions with Arg105 on the mobile loop (Table 4). Even though the initial structure was built to have the mobile loop closed and the guanidinium group of Arg105 in close proximity with the nicotinate, it eventually moved away from 1E4. The absence of this interaction led to loop opening (Table 3) and larger fluctuations in the mobile loop region than those in LDHA:0SN and LDHA:PYR-NADH (Figure 4). These are consistent with the crystal structure of 1E4 in complex with rabbit LDHA, which has the mobile loop either missing (chains A, B, C, G, and H) or open (chains D, E, F), [19] indicative of large mobility and a preference towards the open conformation. On the other hand, 0SN demonstrated marginally better ability to stabilize the LDHA binding site than the native PYR-NADH (Figure 4), which is probably a result of its strong polar interactions with various binding site residues (Tables 4 and S2).

### A-site Binders

**Figure 6:**
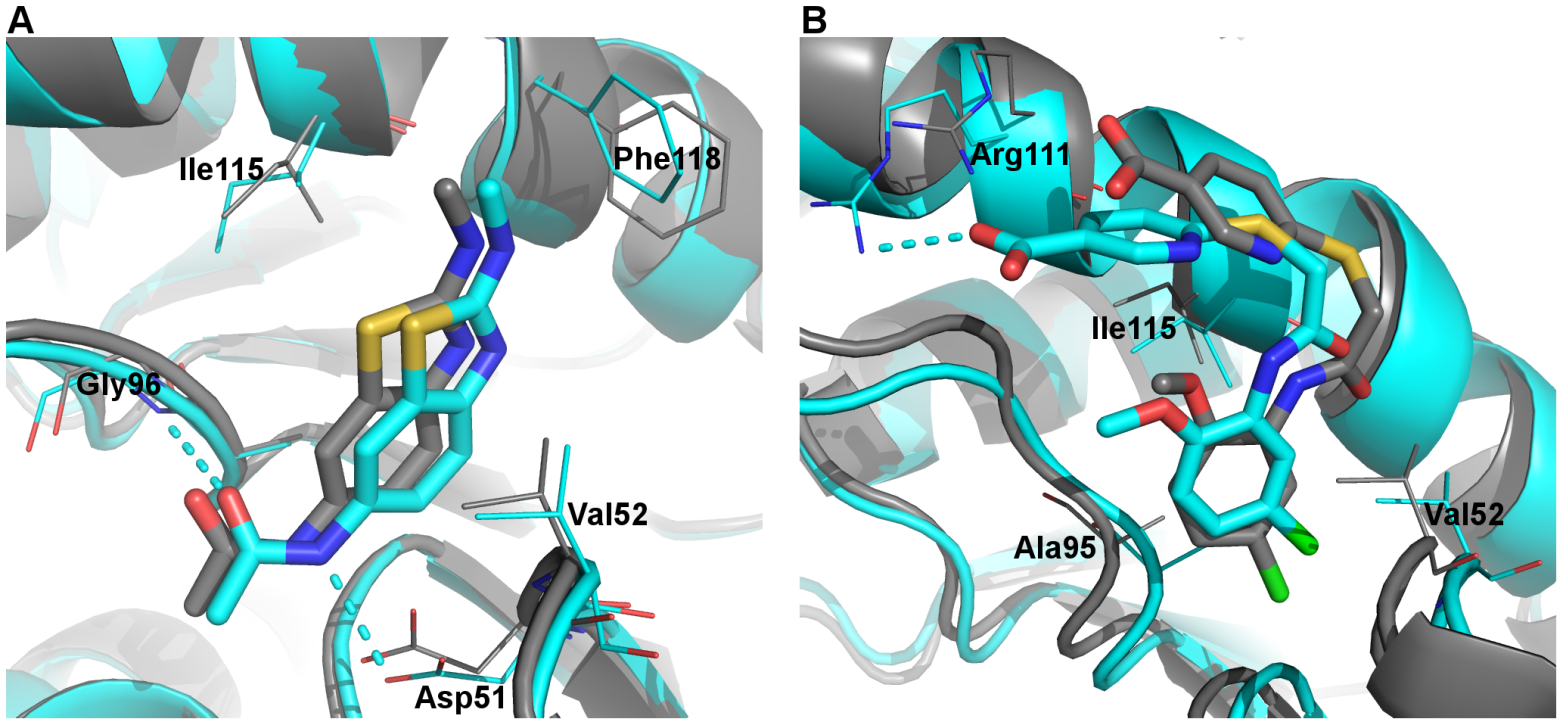
Binding of AJ1 and 1E7. Representative MD structures (cartoon and carbon atoms in cyan) and corresponding crystal structures (cartoon and carbon atoms in grey) are overlaid for A) LDHA:AJ1 and B) LDHA:1E7. Selected binding site residues are labeled and shown in thin lines, while ligands are shown in thick sticks. Dashed lines represent polar interactions. Other atoms are colored: oxygen, red; nitrogen, blue; sulfur, yellow; chlorine, green.

**Figure 7:**
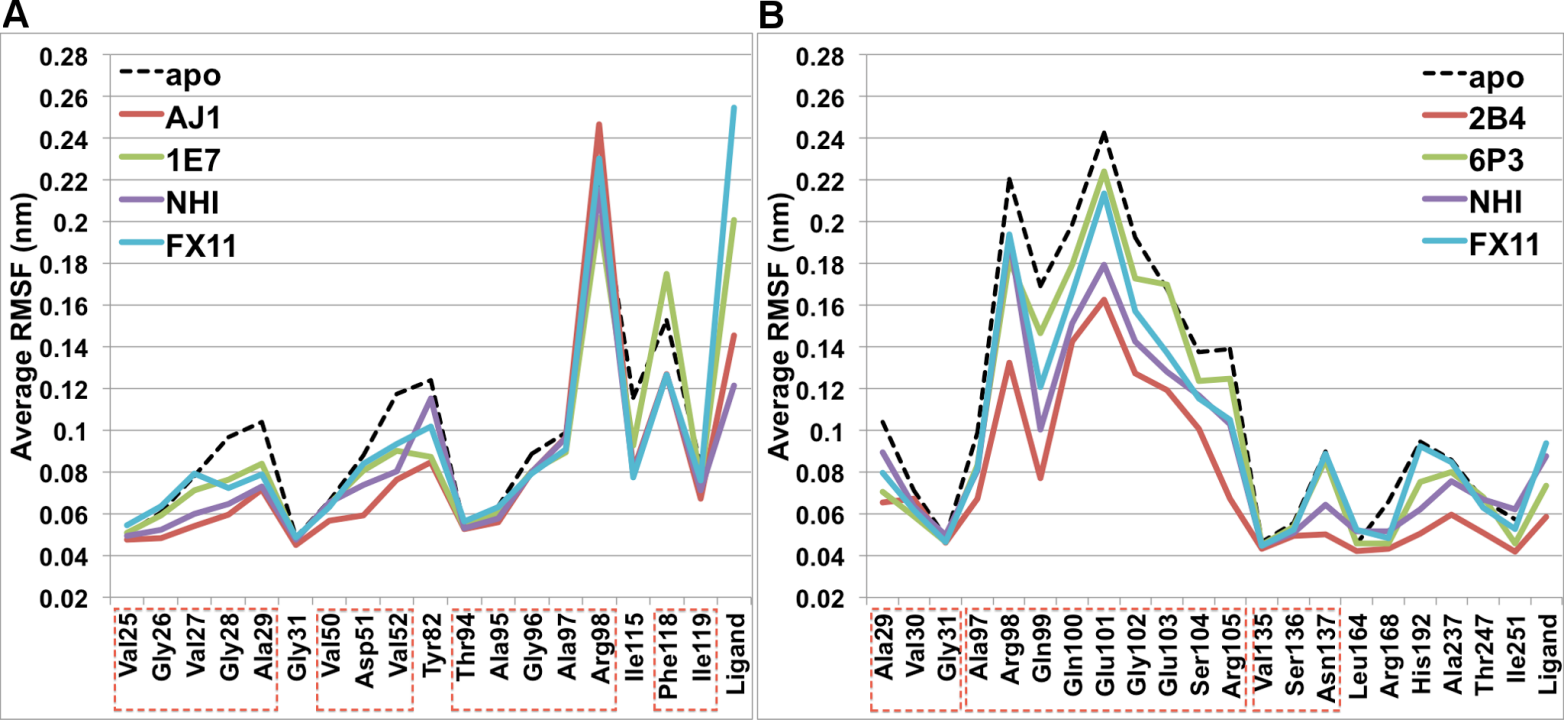
Root-mean-squared fluctuations (RMSF) of A-site binding and S-site binding systems. Contiguous residues are labeled by boxes with red dashed lines.

The binding modes of AJ1 are very similar to the A-end of 0SN. [18] The representative structure from MD simulations was almost identical to the crystal structure, with the only difference being that the phenyl ring of Phe118 moved towards the terminal methyl group of AJ1 to establish hydrophobic interactions (Figure 6A). The benzothiazole ring of AJ1 stayed firmly within the hydrophobic pocket formed by Val52, Ala95, and Ile115, whereas two hydrogen bonds with Asp51 and Gly96 were mostly maintained (Table S2). Nonetheless, these two hydrogen bonds showed lower existence than those in LDHA:0SN, indicating larger mobility than 0SN, which is also in accordance with their relative RMSF values (Figure 7A). Likewise, the binding modes of 1E7 are similar to the A-end of 1E4, [19] although its position and orientation deviated slightly from the corresponding crystal structure during MD simulations (Figure 6B). However, its ionic interactions with Arg111 were not as stable as those in the LDHA:1E4 system (Table 4), and they were established and lost several times during the course of LDHA:1E7 MD simulations. Consequently, 1E7 demonstrated much larger mobility than AJ1 (Figure 7A).

The initial structures of LDHA:NHI_A_ and LDHA:FX11_A_ were constructed by docking the ligand into the A-site of human LDHA, and docking poses with the best overlap between ligand aromatic portions and NADH adenine ring were selected. As expected, both systems evolved into conformations considerably different from docking poses after MD simulations (Text S4).

**Figure 8:**
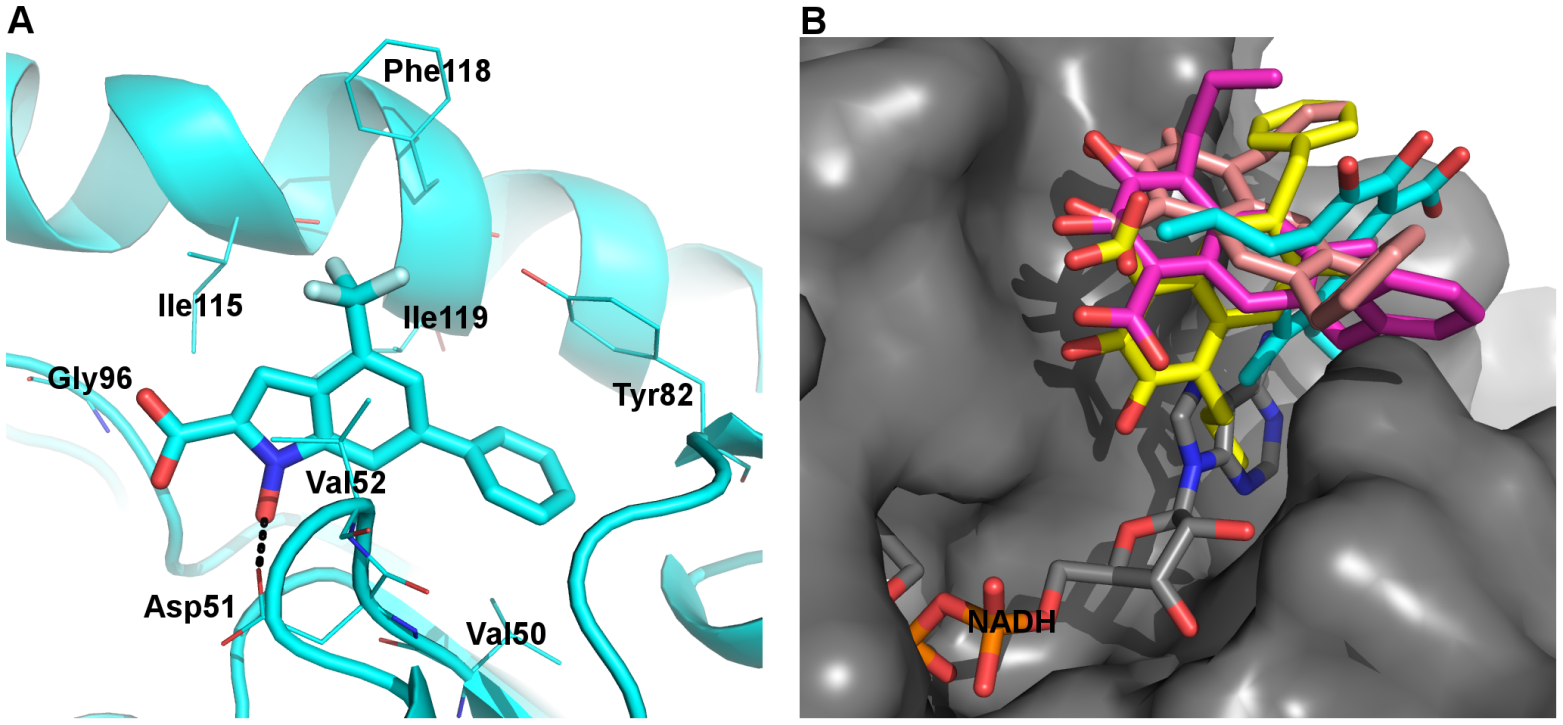
Binding of NHI and FX11 at the A-site. A) A representative MD snapshot of LDHA:NHI_A_, with coloring scheme identical to Figure 6. Black dashed lines represent polar interactions. B) Overlay of representative MD structures from four monomers of LDHA:FX11_A_ (carbon atoms in cyan, magenta, yellow, and pink, respectively) and PDB 1I10 (carbon atoms in grey). Grey surface indicates the solvent-accessible surface of LDHA, showing that two of the FX11 structures are completely outside the binding groove.

Similar to the aromatic ring of other ligands within the A-site, the indole ring of NHI was flanked by two hydrophobic side chains from Val52 and Ile115 (Figure 8A). Its trifluoromethyl moiety was also involved in hydrophobic interactions with Ile115 and Phe118, consistent with inhibition data which shows that the absence of a trifluoromethyl moiety leads to increased *K*_i_ value. [15] The 6-phenyl group is also known to contribute to NHI binding, [15] and it did establish hydrophobic interactions with Val50, Tyr82, and Ile119 during MD simulations. In addition to occasional ionic contacts with Arg98 (Table 4), the carboxylate group of NHI could accept a hydrogen bond from Gly96 N, while its hydroxyl group donated a hydrogen bond to Asp51 (Figure 8A and Table S2). Notably, NHI also exhibited the least mobility among A-site binders modeled (Figure 7A). Hence, NHI might bind to the A-site, in agreement with preliminary crystallographic and NMR data [18].

Unlike NHI, FX11 showed much larger fluctuations in the LDHA:FX11_A_ system, the largest among binders within the A-site (Figure 7A). The representative structures from different monomers displayed large variations, and they were mostly outside the binding groove (Figure 8B). In addition, there was little polar interaction between FX11 and the enzyme (Tables 4 and S2). The collective results suggest that FX11 does not bind within the A-site and behaves more like an unspecific inhibitor, binding near the A-site and not directly competing with NADH binding.

### S-site Binders

As revealed by relevant crystal structures, 2B4 shares the binding patterns of 0SN while 6P3 shares those of 1E4 in the S-site. [18], [19] This was also the case in our MD simulations. Both 2B4 and 6P3 exhibited small fluctuations (Figure 7B) even though the loop opened in the LDHA:6P3 complex and their binding modes were somewhat different. Ionic interactions between the di-carboxylate of 2B4 and the three positively charged S-site residues, Arg105, Arg168, and His192, were persistent during MD simulations (Table 4). Hydrogen bonds donated by Asn137 ND2 and Thr247 OG1 also showed high percentages of existence in the MD trajectory of LDHA:2B4, although the hydrogen bond donated by Gln99 was less frequently present than that in LDHA:0SN (Table S2). The ionic interactions in LDHA:6P3 were mostly between the carboxylate of 6P3 and Arg168 (Table 4), whereas Thr247 OG1 could donate a hydrogen bond to one of the carboxylate oxygens (Table S2). In contrast to 1E4, however, the pyridine ring in 1E7 did not flip to form a hydrogen bond with Asn137 (Text S4).

**Figure 9:**
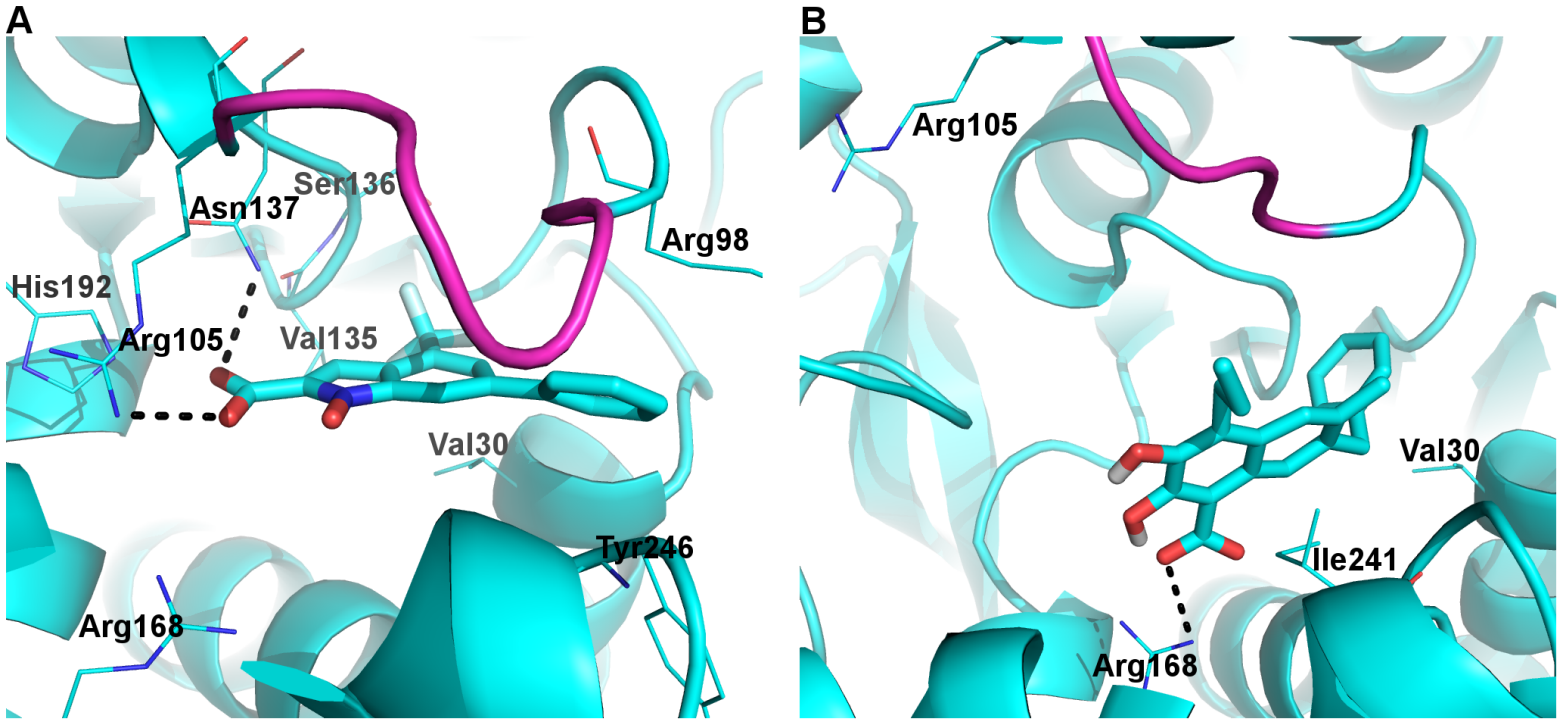
Binding of NHI and FX11 at the S-site. Representative MD snapshots of A) LDHA:NHI_S_ and B) LDHA:FX11_S_ are shown. The color scheme is identical to Figure 6 with hydrogen atoms in white, while the mobile loop is in magenta.

The bound conformation of NHI within the S-site from the MD simulations (Figure 9A) is similar to that previously modeled. [15] The 6-phenyl group is involved in lipophilic interactions with the hydrophobic part of Arg98 and Tyr246, in accordance with its contribution to NHI binding. [15] The trifluoromethyl group sat in a hydrophobic pocket formed by Val30, Val135, and Ser136, also in agreement with experimental data. However, our simulations showed that the carboxylate group was more likely to have ionic interactions with Arg105 than Arg168 (Table 4), and that hydrogen bonding interactions with Asn137 ND2 and Gln99 OE1/NE2 were more frequent than with Thr247 OG1 (Table S2). These interactions led to retention of the closed conformation for the mobile loop, a key difference between our model and the previous one [15].

In the LDHA:FX11_S_ complex, FX11 displayed smaller fluctuations than in the LDHA:FX11_A_ complex and stayed mostly within the S-site (Figure 7). The 7-benzyl group showed some hydrophobic interactions with Val30, while the naphthalene backbone of FX11 exhibited some lipophilic interactions with Ile241 (Figure 9B). Ionic interactions between its carboxylate and Arg168 were mostly maintained (Table 4). Yet, the absence of strong interactions with Arg105 or any other mobile loop residues led to loop opening. Further, its aliphatic chain at C4 was not involved in any significant hydrophobic interactions, while the two hydroxyl groups were mostly involved in intra-molecular hydrogen bonding, not contributing to FX11 binding at all (Figure 9B). Thus, our MD model for LDHA:FX11_S_ is unlikely to represent a reasonable binding mode for the strongest inhibitor (Table 1).

### Steered MD Simulations

It is difficult to discern inhibitors of different binding strengths by conventional MD simulations alone. One alternative approach to achieve discrimination is steered MD, i.e. pulling enzyme inhibitors from the binding site (bound state) to the bulk solvent (unbound state). This has been employed to investigate the relative binding strengths of various enzyme inhibitors. [25], [26] Hence, we performed steered MD simulations in an attempt to differentiate *in silico* the relative binding strengths of LDHA inhibitors at each site of binding. The initial structure for each system was generated from clustering of conventional MD trajectories, and 12 replicate steered MD runs were carried out for each system. Ideally, the difficulty in pulling inhibitors into the bulk solvent should correlate with their binding strengths. This should also help determine the binding location of NHI, as conventional MD simulations of both LDHA:NHI_A_ and LDHA:NHI_S_ systems have generated reasonable binding modes. Based on experimental binding constants (Table 1), it should be more difficult to unbind NHI, from either the A-site or the S-site, than other inhibitors known to bind to the same site. Likewise, it should be most difficult to unbind FX11 if the binding models from conventional MD simulations represent its experimental binding modes.

**Figure 10:**
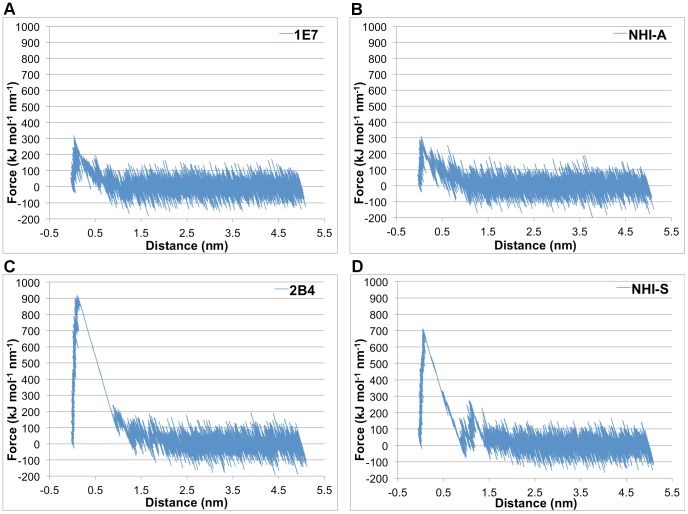
Examples of force-distance curves for the pulling simulation. One of the 12 replicate steered MD runs is shown for A) LDHA:1E7, B) LDHA:NHI_A_, C) LDHA:2B4, and D) LDHA:NHI_S_.

The pulling force as a function of pulling distance was plotted (Figure 10), and the work required to pull the inhibitor out of the binding site was also calculated by integration (Table 5). Pulling A-site binders turned out to be much easier than S-site binders in spite of their comparable binding affinities. This is probably caused by the need to dissociate more interactions and overcome more steric clashes when pulling S-site binders, especially 2B4 and NHI, whose binding kept the mobile loop closed. To demonstrate the influence of different initial loop conformations on the pulling of S-site binders, 6P3 was pulled from two different representative structures, one with the mobile loop open and the other closed (Table 5). As expected, starting from the open conformation required much smaller peak force and less work than starting from the closed conformation. Conversely, pulling 2B4 from two slightly different representative structures, both of which have the mobile loop closed, resulted in a similar peak force and almost identical amount of work (2B4 A and 2B4 B in Table 5). Thus, both the site of binding and the initial conformation of the mobile loop can affect the difficulty of unbinding LDHA inhibitors.

**Table 5:** Work and force involved in the pulling of LDHA binders from their binding sites.

Ligand		ΔG_dissoc_ (kJ mol^−1^)^a^	Work (kJ mol^−1^)^b^	Peak Force (kJ mol^−1 ^nm^−1^)^b^
A-site	**AJ1**	17.8	97.0±19.4	348±29
	**1E7**	22.0	94.4±11.5	347±26
	**NHI**	28.8	126±22	385±65
	**FX11**	41.7	124±20	398±49
S-site	**6P3, loop open**	15.1	169±28	392±48
	**6P3, loop closed**	15.1	575±55	839±86
	**2B4 A**	21.0	679±60	1026±66
	**2B4 B**	21.0	678±91	903±106
	**NHI**	28.8	437±40	778±41
	**FX11**	41.7	207±27	454±49
Dual-site	**0SN**	40.1	806±75	888±66
	**1E4**	40.9	613±55	625±59

^a^ Calculated according to ΔG = −RTln(K_d_) from experimental K_d_ values.

^b^ Reported as average ± standard deviation from 12 replicate steered MD runs.

Regardless of the loop conformation, it took less work and smaller peak force to dissociate 6P3 than 2B4, suggesting that 2B4 is indeed a stronger binder than 6P3. More importantly, the work performed to unbind NHI is much less than that of 2B4 and 6P3 when pulling from the loop-closed conformation, contradicting their relative experimental binding affinities (Table 5). This suggests that the S-site is not the preferred binding site for NHI. The dissociation of FX11, whose binding kept the mobile loop open during conventional MD simulations, turned out to be more difficult than 6P3 when starting from the loop-open conformation. Thus, it appeared that FX11 could bind within the S-site and is indeed a stronger inhibitor than 6P3. Yet, it should be noted that their initial loop conformations are different. The mobile loop in LDHA:FX11_S_ complex is “more closed” than that in LDHA:6P3 (Text S7), and it should be more difficult to unbind FX11 than 6P3 even if they have similar binding affinities within the S-site.

The initial loop conformation had a similar impact on the pulling of both dual-site inhibitors. With the mobile loop being initially closed, the pulling of 0SN required more work and larger peak force than that of 1E4, even though 0SN is a slightly weaker inhibitor (Table 5). Additionally, the work spent on pulling dual-site inhibitors is larger than the combined values of their single-site counterparts (0SN in comparison with AJ1+2B4; 1E4 in comparison with 1E7+6P3, loop open), indicating that the linker moiety in both dual-site inhibitors contributes to their binding.

Within the A-site, neither the pulling work nor the peak force was able to differentiate between the binding strengths of AJ1 and 1E7 (Table 5). Yet, this should be acceptable considering the small difference in their respective experimental ΔG_dissoc_ values and the different experimental conditions under which their binding affinities are measured. [18], [19] In addition, it took statistically more work to pull out NHI than both AJ1 and 1E7 (*p*<0.01), implying that NHI may indeed be an A-site binder. Further, the work and peak force for pulling FX11, which was mostly outside the A-site, are similar to those of NHI. This suggests that they have comparable binding strengths, contrary to the much larger experimental binding affinity of FX11 (Table 1).

Since work is a path-dependent function, it does not necessarily correspond to ΔG_dissoc_, a state function. Thus, the difference in the potential of mean force (ΔPMF) was calculated using the Jarzynski equality. [27], [28] This equates the exponential average of the work performed during a non-equilibrium process, such as the pulling process of our steered MD simulations, to ΔPMF, which is a state function and equal to the free energy difference, if a sufficient number of replicates are used in the calculation:

exp(−ΔPMF/*k*_B_T) = <exp(−W/*k*_B_T)>

In the Jarzynski equality, W is the work performed, *k*_B_ is the Boltzmann constant, and T is the temperature in Kelvin. The angled brackets indicate an average of the exponential over all replicate runs. With insufficient number of replicate steered MD runs, it is expected that the ΔPMF obtained will overestimate the corresponding ΔG_dissoc_ values, [29], [30] which is indeed the case in our calculations (Table 6). Notwithstanding the significant overestimation, our ΔPMF values reproduced the experimental order of binding strengths for A-site binders AJ1, 1E7, and NHI as well as S-site binders 6P3 and 2B4. Furthermore, the differences between ΔPMF values (ΔΔPMF) show qualitative correlation with the corresponding ΔG_dissoc_ differences (ΔΔG_dissoc_) for A-site binders (except NHI vs FX11), the pulling of which were not complicated by different mobile loop conformations. Again, this suggests that NHI binds in the A-site.

**Table 6:** Comparison of experimental binding free energies and calculated ΔPMF.

Ligand		ΔG_dissoc_ (kJ mol^−1^)^a^	ΔPMF (kJ mol^−1^)	ΔΔG_dissoc_ (kJ mol^−1^)^b^	ΔΔPMF (kJ mol^−1^)^b^
A-site	**AJ1**	17.8	72.7	0	0
	**1E7**	22.0	76.5	4.2	3.8
	**NHI**	28.8	95.0	11.0	22.3
	**FX11**	41.7	92.4	23.9	19.7
S-site	**6P3, loop open**	15.1	117.8		
	**6P3, loop closed**	15.1	449.7		
	**2B4, A**	21.0	564.1		
	**2B4, B**	21.0	546.5		
	**NHI**	28.8	372.2		
	**FX11**	41.7	181.7		
Dual-site	**0SN**	40.1	687.4		
	**1E4**	40.9	526.6		

^a^ Calculated according to ΔG = −RTln(K_d_) from experimental K_d_ values.

^b^ Calculated by subtracting the ΔG_dissoc_ or ΔPMF of AJ1 for A-site binders.

## Discussion

The use of a tetrameric model to study LDHA computationally has been attempted previously. [31], [32] However, those studies were based on evidence from either geometry optimization or short-term MD simulations with restraints to prevent large conformational changes. [31], [32] In contrast, the present study employed moderate-length MD simulations with sufficient system size (>150,000 atoms) and no restraints to approximate physiological conditions, further justifying the use of the tetrameric form in such computational studies. Of note, LDHAs from different species (Figure S1) might show different dynamics. However, we restricted this study to human LDHA, which is most relevant to the development of anticancer agents; only 0SN has been co-crystalized with human LDHA among the ligands studied (Table 1).

We have shown that the mobile loop prefers to be in an open conformation for most of the LDHA:ligand systems investigated (Table 2), leaving the S-site exposed to the bulk solvent. Three systems, LDHA:0SN, LDHA:2B4, and LDHA:NHI_S_, could hold the mobile loop in the closed conformation. Additionally, the mobile loop displayed larger fluctuations in the open conformation than in the closed conformation, which is probably caused by a much larger conformational space available for the loop open state. It follows that bringing the mobile loop to the closed conformation causes an entropic penalty. This could partially explain the comparable binding affinities of 0SN and 1E4, even though 0SN possesses more polar interactions.

Similarly, the ionic interactions with Arg111 were shown to significantly reduce the mobility of 1E4 and surrounding A-site residues, including Arg111; the incurred entropic penalty would offset the enthalpy gain from such strong ionic interactions. Since Arg111 is largely exposed to bulk solvent, polar water molecules can also compete with the inhibitor in interacting with Arg111. Notably, similar ionic interactions in the LDHA:1E7 complex appeared to be unstable, suggesting little free energy gain from this interaction.

No significant correlation between the dynamics of ligand binding, as revealed by RMSF values of binding site residues and ligands as well as the percentage existence of polar interactions, and experimental binding affinities was found. For example, the binding of 1E4 incurred much larger fluctuations with smaller percentage existence of polar interactions than that of 0SN (Figure 4, Tables 4 and S3), but their experimental binding affinities are roughly the same, with 1E4 being slightly higher (Table 1). The same phenomenon was observed for A-site binders 1E7 and AJ1. Likewise, the number of strong polar interactions or contacts (Table S1) does not predict the strength of binding. Hence, conventional MD simulations appear to be incapable of discriminating LDHA inhibitors of different binding strengths. To resolve this issue, we resorted to steered MD simulations, which can qualitatively discern inhibitors of largely different binding affinities [33].

Steered MD simulations have demonstrated the effects of different initial conformations of the mobile loop (6P3, loop open vs 6P3, loop closed) and different sites of binding (6P3, loop open vs AJ1/1E7) on the difficulty of pulling. Considering these effects, our pulling results correlated well with experimental binding affinities and were able to distinguish inhibitors with a small 4 kJ mol^−1^ ΔG_dissoc_ difference, despite their different dynamics and modes of binding (Table 6). Although ΔPMF values, calculated from exponential averages of non-equilibrium work, largely depend on rarely sampled trajectories with small dissipated work (Text S6), the work and peak force were able to qualitatively discriminate inhibitors of the same binding site and initial loop conformation (for S-site binders). Other computational approaches such as umbrella sampling can yield a better estimate of free binding energy. [34], [35] Nevertheless, steered MD simulations provide a more convenient set-up with much less computational cost for ranking inhibitors with respect to relative binding affinities.

Our steered MD simulations also suggest that NHI is more likely to bind in the A-site by comparison of relative difficulties in pulling, even though NHI binding models in both the A-site and the S-site, generated from conventional MD simulations, can explain its experimental structure-activity relationships. [15] After all, NHI behaved differently in the S-site from other inhibitors that have only one carboxylate group within the S-site, in that NHI could hold the mobile loop closed by interacting with Arg105 for most of the time while others could not (Tables 2 and 4). The binding of NHI at the A-site also agrees with preliminary NMR and crystallographic data. [18] On the other hand, our attempts to obtain possible binding modes of FX11 were unsuccessful. In its A-site binding models (Figure 8B), only the propyl group is within the A-site while the naphthalene backbone is mostly outside. In addition, steered MD results suggest that FX11 would have a similar binding affinity to NHI if it binds around this site, which contradicts their experimental binding data (Table 1). Moreover, pulling results cannot be used to support FX11 binding at the S-site due to the incomparability incurred by different loop conformations between FX11 and 6P3, loop open (Text S7). Yet, the lack of important interactions (Figure 9 and Table S2) does indicate weak binding of FX11 with the S-site. All these observations are consistent with recent literature that suggests the super-stoichiometric and unspecific binding of FX11 due to its aggregation instead of binding at a specific site [19].

In light of the interaction dynamics of LDHA:ligand systems, the design of stronger LDHA inhibitors could benefit from introducing contacts with binding site residues that are intrinsically stable, which could be inferred from their RMSF values in the simulation of apo LDHA (Figure 4). For A-site binders, hydrophobic contacts with Val50, Ala95, and Ile119, all of which are indicated in our NHI binding model, would be most recommended. Involving Arg98 and/or Arg111 in ionic interactions may not be optimal, as they showed large RMSF values in apo LDHA and even some LDHA:ligand simulations. Neither 0SN nor 1E4 has polar interactions with Arg98, but they are stronger binders than NADH (Table 1), whose binding greatly reduced the mobility of Arg98 (Figure 4) and presumably incurred a large entropic penalty. Yet, novel A-site inhibitors could be designed to exploit ionic interactions with Asp51, which serves as an important and stable hydrogen bond acceptor for most binders in this study (Table S2). For example, introducing a positively charged group at the para-position of the phenyl ring in 1E7 could enhance its binding affinity. Additionally, polar interactions with Thr94 and Gly96 could also be incorporated in the design of A-site inhibitors. For S-site binders, hydrophobic interactions with Val135 and Ile251, which are deep under the binding site and exhibited very small fluctuations, should be considered in addition to Val30. To this end, a methyl group could be attached to the aromatic rings of S-site inhibitors. Ionic contacts with Arg168 and His192 are apparently necessary, while hydrogen bonding interactions with Asn137 and Thr247 should also be maintained. Interactions with mobile loop residues would be less favorable as there would be considerable entropic costs in stabilizing these residues.

## Conclusions

We have conducted conventional MD simulations to investigate several LDHA inhibitors with known sites of binding, which revealed different binding dynamics for inhibitors of similar binding affinities. In addition, the binding location and geometry of two inhibitors, NHI and FX11, whose binding sites were subject to question, were also probed. This resulted in two possible binding models for NHI and two unlikely ones for FX11, the latter of which agrees with recent literature showing the unspecific binding of FX11. To differentiate LDHA inhibitors in terms of binding strengths, steered MD simulations were performed to pull inhibitors out of the binding site. Good correlation between the difficulties of unbinding inhibitors, especially as measured by ΔPMF values calculated from the Jarzynski equality, and experimental ΔG_dissoc_ values was achieved. The pulling results also suggest the favorable binding of NHI within the A-site instead of the S-site, consistent with experimental data.

The combined use of conventional and steered MD simulations as presented herein could be applied to newly-designed LDHA inhibitors, so that their binding modes and strengths relative to known inhibitors of the same binding site could be inferred prior to chemical synthesis and biological evaluation. This approach would assist in the design and development of better LDHA inhibitors, contributing to the growing efforts that target energy metabolism for cancer therapy.

## Materials and Methods

### System Preparation

Two crystal structures of human LDHA [17], [18] (PDBs 1I10 and 4AJP) have been used in MD simulations, and their structures are very similar (Table S3). Prior to MD simulations, they were submitted to MolProbity [36] for the addition of hydrogen atoms and validation of structures. The LDHA:0SN system was constructed directly from the PDB 4AJP complex after removal of glycerol and sulfate ions, and the initial structure of LDHA:1E4 was built by fitting 1E4 (from chain A of PDB 4I9H) analogously into the four binding sites of LDHA:0SN complex after removing 0SN. The starting structure of the LDHA:PYR-NADH system was built by altering the oxamate in PDB 1I10 to pyruvate. The initial structures of LDHA:1E7, LDHA:AJ1, LDHA:2B4, and LDHA:6P3 complexes were constructed by fitting the ligand structure (from chain A of PDBs 4I9U, 4AJ1, 4AJE, and 4I8X, respectively) analogously into the binding sites of human LDHA from PDB 1I10 (chains A, B, C, and D). To build the starting structure for LDHA:FX11 and LDHA:NHI complexes, molecular docking was performed in both the adenine and the substrate/nicotinamide binding pockets by AutoDock Vina. [37] After removing all non-protein residues, chain A (loop closed) and chain D (loop open) of PDB 1I10 was used as the macromolecular model for the docking into the adenine pocket and substrate/nicotinamide pocket, respectively. The docking poses with the best overlap with NADH adenine (as in PDB 1I10 chain D) were selected for adenine pocket docking, while those having the best interaction between ligand carboxylate and Arg168 guanidinium group were chosen for substrate/nicotinamide pocket docking. For apo LDHA, the starting structure was directly extracted from PDB 1I10 (chains A, B, C, and D) after removal of non-protein residues. Hence, initial structures of 12 systems were built (Table 2).

All complex and apo LDHA structures were processed by the LEaP module in AmberTools1.5, [38] where Amber ff99SB [39] and GAFF force field [40] parameters were applied. Parameters for NADH were taken directly from R.E.D. Database Project F-90. [41] Missing atomic charges for ligands were derived by R.E.D. Server [42] according to the RESP model, [43] taking into account multiple conformations and multiple orientations as per AMBER convention (Text S1) [44].

### Conventional MD Simulations

MD simulations of all systems were conducted with GROMACS 4.5.4. [45] Each system was placed in a dodecahedral box with a minimal 1.5 nm distance between solute and box edge, followed by solvation with TIP3P water molecules. Salt ions were then added at a concentration of 0.15 M to balance ionic charge in the system. Energy minimizations were carried out with steepest descent integrator and conjugate gradient algorithm sequentially to achieve a maximum force of less than 500 kJ mol-1 nm-1 on any atom. A twin-range cutoff scheme was used to evaluate short-range, non-bonded interactions, with van der Waals interactions truncated at 1.4 nm and electrostatic interactions truncated at 0.9 nm. Long-range electrostatic interactions were treated by the particle mesh Ewald (PME) method. [46], [47] The temperature was maintained at 298 K using a velocity rescaling thermostat [48] with a coupling constant of 0.1 ps, while the pressure was maintained at 1.0 atm using a Berendsen barostat, [49] with a coupling constant of 1 ps. Simulations were performed with a time step of 2 fs, and all bonds involving hydrogen atoms were constrained by a parallel linear constraint solver (P-LINCS). [50] Following equilibration under a constant volume (NVT) ensemble for 100 ps, three stages of equilibrations under a constant pressure (NPT) ensemble were carried out for 100 ps, 100 ps, and 300 ps, respectively, reducing the force constant of harmonic position restraint applied on system heavy atoms from 1000 kJ mol^−1^ nm^−2^, 300 kJ mol^−1^ nm^−2^, to 100 kJ mol^−1^ nm^−2^, respectively. For LDHA:FX11 and LDHA:NHI systems, no position restraints were applied on the ligand and enzyme atoms within 0.5 nm of the ligand. After equilibration, production MD simulations were conducted for 60 ns for each system without any constraints. Three replicate MD runs were performed for each system by varying the random seed for initial velocity generation, resulting in a total simulation time of 2.16 µs.

### Trajectory Analysis

The last 20 ns of each triplicate trajectory was extracted and combined, yielding 60 ns of equilibrated trajectory for each system. Clustering was then performed for each monomer based on RMSD values of heavy atoms from the ligand and selected binding site residues, the latter of which include LDHA residues within 0.5 nm of 0SN in chain A of PDB 4AJP (Text S3). The gromos clustering method was employed with a certain RMSD cutoff so that the largest cluster represent more than half of the conformations of the 60 ns combined trajectory. For each system, the central structure of the largest cluster in the monomer was chosen as the representative structure. Except for RMSD, all other analyses were performed on the combined 60 ns trajectory.

### Steered MD Simulations

The representative structure of the monomer with the least RMSF values was used as the starting point of the non-equilibrium pulling for each system (Text S5). The corresponding tetramer was solvated with TIP3P water and 0.15 M salt ions in a 20 nm×11 nm×11 nm rectangular box, whose dimension is more than sufficient for the pulling simulation. This resulted in a simulation system size of ∼240,000 atoms. After equilibration under a NPT ensemble for 100 ps, the pressure and heat controls were switched to a Nose-Hoover thermostat [51] and a Parrinello-Raham barostat [52] and only the ligand in the representative monomer was pulled away. Harmonic position restraints with a force constant of 1000 kJ mol^−1^ nm^−2^ were applied on heavy atoms of the other three ligand residues and LDHA residues. In the case of pulling S-site binders, no position restraints were applied on the mobile loop region (residues 95–112) to allow for its movement and thus the egress of ligands. Constant-velocity steered MD simulations were implemented with a spring force constant of 1000 kJ mol^–1^ nm^–2^ at a relatively low speed of 0.5 nm ns^–1^. The pulling force and distance were output every 1 ps to a total pulling time of 10 ns, and the force-distance curve consisting of 10,000 points were numerically integrated to yield work. As the work performed by non-equilibrium pulling processes is strongly path-dependent, the direction of the pulling force was dynamically defined for each individual system. Initially, at least three different pulling directions were selected manually to minimize steric clashes, and the one producing the least amount of work was used for subsequent duplicate pulling simulations. A total of 12 replicate pulling simulations were performed for each system, totaling a simulation time of 1.44 µs.

All simulations were performed on a parallel HP Xeon E5649 cluster provided by Compute Canada. Each conventional and steered MD job ran with 144 CPU cores and the total simulation time was estimated to be 100 core years.

## Supporting Information

Figure S1 **Sequence alignment of lactate dehydrogenase A (LDHA) from different species.** All sequences were retrieved from http://www.uniprot.org/. Residue numbering is one larger than that in the manuscript, as the initiator methionine was counted here. The symbol in the last row of each column indicates whether the residues are identical (*), strongly similar (:), or weakly similar (.).
(PDF)

Table S1 **Averaged number of contacts between LDHA and the ligand.**
(PDF)

Table S2 **Hydrogen bond (excluding ionic interactions) occupancy.**
(PDF)

Table S3 **Root mean squared deviations (RMSD) between PDB 1I10 and PDB 4AJP.**
(PDF)

Text S1 **Atom names, types, and RESP charges of LDHA ligands.**
(PDF)

Text S2 **Root mean squared deviation (RMSD) of LDHA backbone atoms.**
(PDF)

Text S3 **Root mean squared deviation (RMSD) of heavy atoms of selected binding site residues and ligands.**
(PDF)

Text S4 **Superimposition of cluster centroids.**
(PDF)

Text S5 **Initial structures for steered MD simulations.**
(PDF)

Text S6 **Original pulling work and peak force for steered MD runs.**
(PDF)

Text S7 **Loop conformations for the pulling of S-site inhibitors.**
(PDF)
