# Supplementary material for: Human Lactate Dehydrogenase A Inhibitors: A Molecular Dynamics Investigation
Source: PLoS One. 2014 Jan 17;9(1):e86365. doi: 10.1371/journal.pone.0086365 (PMC3895040; doi:10.1371/journal.pone.0086365)
Supplement: Figure S1 — Sequence alignment of lactate dehydrogenase A (LDHA) from different species. All sequences were retrieved from http://www.uniprot.org/. Residue numbering is one larger than that in the manuscript, as the initiator methionine was counted here. The symbol in the last row of each column indicates whether the residues are identical (*), strongly similar (:), or weakly similar (.). (PDF) [file pone.0086365.s001.pdf]

```
1     MATLKDQLIYNLLKEEQTPQNKITVVGVGAVGMACAISILMKDLADELALVDVIEDKLKG   60   P00338   LDHA_HUMAN
1     MAALKDQLIVNLLKEEQVPQNKITVVGVGAVGMACAISILMKDLADELALVDVIEDKLKG   60   P04642   LDHA_RAT
1     MAALKDQLIHNLLKEEHVPQNKITVVGVGAVGMACAISILMKDLADELALVDVMEDKLKG   60   P13491   LDHA_RABIT
1     MATLKDQLIHNLLKEEHVPHNKITVVGVGAVGMACAISILMKELADEIALVDVMEDKLKG   60   P00339   LDHA_PIG
      **:****** ******:.*:**********************:****:*****:******

61    EMMDLQHGSLFLRTPKIVSGKDYNVTANSKLVIITAGARQQEGESRLNLVQRNVNIFKFI  120   P00338   LDHA_HUMAN
61    EMMDLQHGSLFLKTPKIVSSKDYSVTANSKLVIITAGARQQEGESRLNLVQRNVNIFKFI  120   P04642   LDHA_RAT
61    EMMDLQHGSLFLRTPKIVSGKDYSVTANSKLVIITAGARQQEGESRLNLVQRNVNIFKFI  120   P13491   LDHA_RABIT
61    EMMDLQHGSLFLRTPKIVSGKDYNVTANSRLVVITAGARQQEGESRLNLVQRNVNIFKFI  120   P00339   LDHA_PIG
      ************:******.***.*****:**:***************************

121   IPNVVKYSPNCKLLIVSNPVDILTYVAWKISGFPKNRVIGSGCNLDSARFRYLMGERLGV  180   P00338   LDHA_HUMAN
121   IPNVVKYSPQCKLLIVSNPVDILTYVAWKISGFPKNRVIGSGCNLDSARFRYLMGERLGV  180   P04642   LDHA_RAT
121   IPNVVKYSPHCKLLVVSNPVDILTYVAWKISGFPKNRVIGSGCNLDSARFRYLMGERLGV  180   P13491   LDHA_RABIT
121   IPNIVKYSPNCKLLVVSNPVDILTYVAWKISGFPKNRVIGSGCNLDSARFRYLMGERLGV  180   P00339   LDHA_PIG
      ***:*****.****:*********************************************

181   HPLSCHGWVLGEHGDSSVPVWSGMNVAGVSLKTLHPDLGTDKDKEQWKEVHKQVVESAYE  240   P00338   LDHA_HUMAN
181   HPLSCHGWVLGEHGDSSVPVWSGVNVAGVSLKSLNPQLGTDADKEQWKDVHKQVVDSAYE  240   P04642   LDHA_RAT
181   HALSCHGWILGEHGDSSVPVWSGMNVAGVSLKTLHPELGTDADKEQWKQVHKQVVDSAYE  240   P13491   LDHA_RABIT
181   HPLSCHGWILGEHGDSSVPVWSGVNVAGVSLKNLHPELGTDADKEHWKAVHKQVVDSAYE  240   P00339   LDHA_PIG
      * ******:**************:********.*.*:**** ***:** ******:****

241   VIKLKGYTSWAIGLSVADLAESIMKNLRRVHPVSTMIKGLYGIKDDVFLSVPCILGQNGI  300   P00338   LDHA_HUMAN
241   VIKLKGYTSWAIGLSVADLAESIMKNLRRVHPISTMIKGLYGIKEDVFLSVPCILGQNGI  300   P04642   LDHA_RAT
241   VIKLKGYTTWAIGLSVADLAESIMKNLRRVHPISTMLKGLYGIKEDVFLSVPCVLGQNGI  300   P13491   LDHA_RABIT
241   VIKLKGYTSWAIGLSVADLAESIMKNLRRVHPISTMIKGLYGIKEDVFLSVPCILGQNGI  300   P00339   LDHA_PIG
      ********:***********************:***:*******:********:******

301   SDLVKVTLTSEEEARLKKSADTLWGIQKELQF   332   P00338   LDHA_HUMAN
301   SDVVKVTLTPDEEARLKKSADTLWGIQKELQF   332   P04642   LDHA_RAT
301   SDVVKVTLTSEEEAHLKKSADTLWGIQKELQF   332   P13491   LDHA_RABIT
301   SDVVKVTLTPEEEAHLKKSADTLWGIQKELQF   332   P00339   LDHA_PIG
      **:****** :***:*****************
```
