# Supplementary material for: Human Lactate Dehydrogenase A Inhibitors: A Molecular Dynamics Investigation
Source: PLoS One. 2014 Jan 17;9(1):e86365. doi: 10.1371/journal.pone.0086365 (PMC3895040; doi:10.1371/journal.pone.0086365)
Supplement: Table S1 — Averaged number of contacts between LDHA and the ligand. (PDF) [file pone.0086365.s002.pdf]

Table S1. Averaged number of contacts between LDHA and the ligand.

Ligand		Number of contacts	Number of contacts per ligand heavy atom
Dual site	PYR-NADH	167.1	3.34
	0SN	100.4	2.87
	1E4	98.6	1.93
A-site	1E7	36.7	1.60
	AJ1	35.0	2.33
	NHI	50.9	2.21
	FX11	33.4	1.28
S-site	2B4	56.4	3.76
	6P3	39.3	2.62
	NHI	60.0	2.61
	FX11	52.0	2.00
