# Supplementary material for: Human Lactate Dehydrogenase A Inhibitors: A Molecular Dynamics Investigation
Source: PLoS One. 2014 Jan 17;9(1):e86365. doi: 10.1371/journal.pone.0086365 (PMC3895040; doi:10.1371/journal.pone.0086365)
Supplement: Table S2 — Hydrogen bond (excluding ionic interactions) occupancy. (PDF) [file pone.0086365.s003.pdf]

Table S2. Hydrogen bond (excluding ionic interactions) occupancy.

System	Donor		Acceptor		Occupancy[d]
LDHA:PYR-NADH	ALA29	N	POP[a]	OA2	82.06%
	VAL30	N	POP[a]	OB1	94.67%
	LYS56	NZ	ADS[b]	O3'	52.39%
	ASN137	N	NRS[c]	O3'/O2'	79.22%
	ASN137	ND2	PYR	O	69%
	HIP192	NE2	PYR	O	70.39%
	THR247	OG1	PYR	OX2	67.94%
	ADS[b]	O3'	ASP51	OD2/OD1	100%
	ADS[b]	O2'	ASP51	OD1/OD2	99.56%
	NRS[c]	O3'	ALA97	O	63.44%
	NRS[c]	N9	VAL135	O	84%
LDHA:0SN	GLY96	N	0SN	O13	91.92%
	GLN99	NE2	0SN	O31	71.21%
	ASN137	ND2	0SN	O32/O31	96.37%
	THR247	OG1	0SN	O34	83.96%
	0SN	N11	ASP51	OD2	76.33%
	0SN	N16	GLY96	O	96.04%
LDHA:1E4	GLY96	N	1E4	O17	33.75%*
	ASN137	ND2	1E4	N36	27.33%*
	THR247	OG1	1E4	OY2	25.75%
	1E4	O19	ASP51	OD2	15.17%
	1E4	O20	THR94	O	24.29%
LDHA:AJ1	GLY96	N	AJ1	O	63.45%
	AJ1	N	ASP51	OD2/ OD1	71.15%
LDHA:NHI_A	GLY96	N	NHI	OY2/OY1	25.38%*
	NHI	O1	ASP51	OD1/OD2	41.75%*
	NHI	O1	GLY96	O	19.63%*
LDHA:2B4	GLN99	NE2	2B4	OY1	36%
	ASN137	ND2	2B4	OY2/OY1	96.61%
	THR247	OG1	2B4	OX1	78.56%
LDHA:6P3	THR247	OG1	6P3	OY2/OY1	41.67%
LDHA:NHI_S	ASN137	ND2	NHI	OY1/OY2	38.89%*
	THR247	OG1	NHI	O1	16.89%*
	NHI	O1	GLN99	OE1	20.44%*
LDHA:$FX11_S$	THR247	OG1	FXI	OX1/OX2	45.62%*

[a]The diphosphate moiety of NADH.
[b]Adenosine.
[c]Nicotinamide riboside.
[d]Hydrogen bonds not present in crystal structures are indicated by stars (*).
