# Supplementary material for: Human Lactate Dehydrogenase A Inhibitors: A Molecular Dynamics Investigation
Source: PLoS One. 2014 Jan 17;9(1):e86365. doi: 10.1371/journal.pone.0086365 (PMC3895040; doi:10.1371/journal.pone.0086365)
Supplement: Table S3 — Root mean squared deviations (RMSD) between PDB 1I10 and PDB 4AJP. (PDF) [file pone.0086365.s004.pdf]

Table S3. Root mean squared deviations (RMSD) between PDB 1I10 and PDB 4AJP.

	**Root mean squared deviations (nm)**		
**Chains**	All protein atoms	Backbone atoms	Binding site residues
A	0.088	0.045	0.082
B	0.085	0.046	0.075
C	0.092	0.054	0.070
D	0.190	0.157	0.353
