# Supplementary material for: Human Lactate Dehydrogenase A Inhibitors: A Molecular Dynamics Investigation
Source: PLoS One. 2014 Jan 17;9(1):e86365. doi: 10.1371/journal.pone.0086365 (PMC3895040; doi:10.1371/journal.pone.0086365)
Supplement: Text S1 — Atom names, types, and RESP charges of LDHA ligands. (PDF) [file pone.0086365.s005.pdf]

Text S1. Atom names, types, and RESP charges of LDHA ligands.

PYR[a]	Atom name/type[b]	RESP charge (*e*)
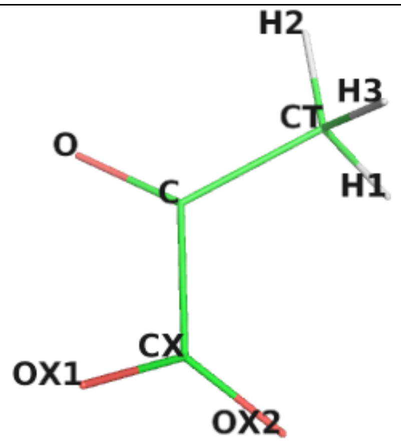	C/C	0.5118
	O/O	-0.5999
	CX/C	0.7279
	OX1/O2	-0.7782
	OX2/O2	-0.7782
	CT/CT	-0.1518
	H1/HC	0.0228
	H2/HC	0.0228
	H3/HC	0.0228

[a]RESP charges of NADH were taken directly from RESP ESP charge DDataBase, Project F-90.[1]
[b]Atom types refer to those in either Amber ff99SB (upper case) or GAFF force field (lower case), the latter of which is an expansion to the former and they are thus compatible.[2]

1. Dupradeau FY, Cezard C, Lelong R, Stanislawiak E, Pecher J, et al. (2008) R.E.DD.B.: a database for RESP and ESP atomic charges, and force field libraries. Nucleic Acids Res 36: D360-367.
2. Wang J, Wolf RM, Caldwell JW, Kollman PA, Case DA (2004) Development and testing of a general amber force field. J Comput Chem 25: 1157-1174.

0SN	Atom name/type	RESP charge (*e*)	Atom name/type	RESP charge (*e*)
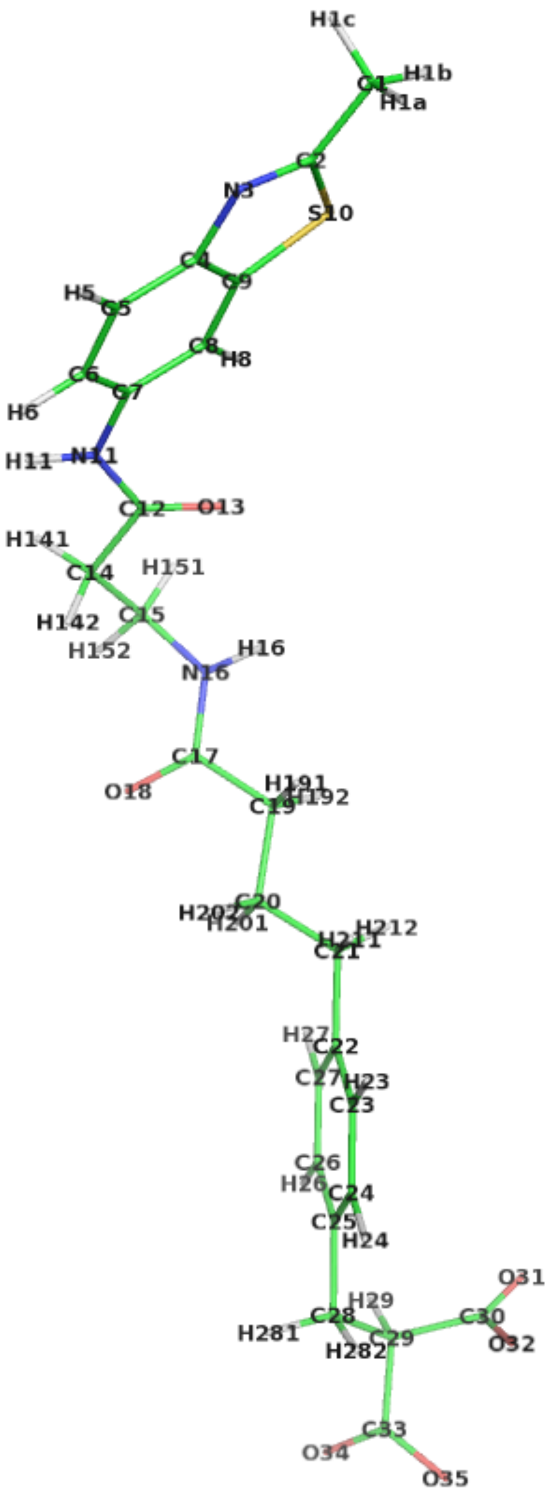	C1/CT	-0.0909	H5/HA	0.1523
	C2/CC	0.3679	H6/HA	0.1684
	N3/NB	-0.5663	H8/HA	0.2406
	C4/CB	0.3426	H11/H	0.2844
	C5/CA	-0.1537	H141/HC	0.0364
	C6/CA	-0.2620	H142/HC	0.0364
	C7/C	0.0513	H151/H1	0.0628
	C8/CA	-0.1189	H152/H1	0.0628
	C9/CB	-0.0907	H16/H	0.3158
	S10/S	-0.1402	H191//HC	0.0989
	N11/N	-0.3850	H192/HC	0.0989
	C12/C	0.6184	H201/HC	-0.0004
	O13/O	-0.5748	H202/HC	-0.0004
	C14/CT	-0.0879	H211/HC	0.0099
	C15/CT	0.0512	H212/HC	0.0099
	N16/N	-0.5023	H23/HA	0.1432
	C115/C	0.6712	H24/HA	0.1074
	O18/O	-0.6262	H26/HA	0.1074
	C19/CT	-0.3546	H27/HA	0.1432
	C20/CT	0.0799	H281/HC	-0.0628
	C21/CT	0.0316	H282/HC	-0.0628
	C22/CA	-0.0109	H29/HC	-0.1944
	C23/CA	-0.3116	H1A/HC	0.0553
	C24/CA	-0.0753	H1B/HC	0.0553
	C25/CA	0.0899	H1C/HC	0.0553
	C26/CA	-0.0753		
	C27/CA	-0.3116		
	C28/CT	0.0187		
	C29/CT	0.3317		
	C30/C	0.6900		
	O31/O2	-0.8050		
	O32/O2	-0.8050		
	C33/C	0.6900		
	O34/O2	-0.8050		
	O35/O2	-0.8050		

2B4	Atom name/type	RESP charge (*e*)
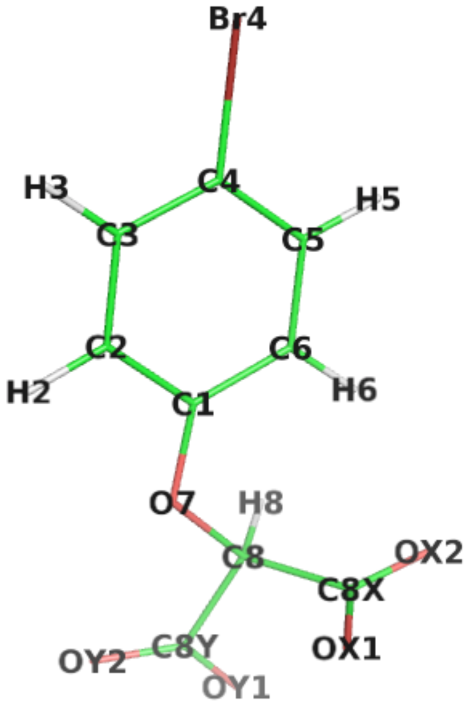	C4/ca	-0.0745
	BR4/br	-0.2384
	C3/ca	-0.1105
	H3/ha	0.1338
	C2/ca	-0.2691
	H2/ha	0.1458
	C1/ca	0.5431
	C6/ca	-0.2691
	H6/ha	0.1458
	C5/ca	-0.1105
	H5/ha	0.1338
	O7/os	-0.5457
	C8/c3	0.3328
	H8/h1	-0.0955
	C8X/c	0.7727
	OX1/o	-0.8168
	OX2/o	-0.8168
	C8Y/c	0.7727
	OY1/o	-0.8168
	OY2/o	-0.8168

AJ1	Atom name/type	RESP charge (*e*)
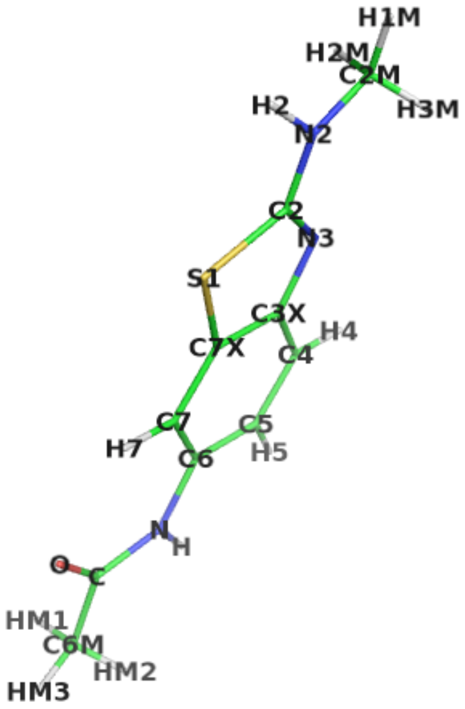	C3X/ca	0.3851
	C7X/ca	-0.0434
	C7/ca	-0.1932
	H7ha	0.2225
	C6/ca	0.0005
	C5/ca	-0.1238
	H5/ha	0.1626
	C4/ca	-0.2805
	H4/ha	0.1712
	N/n	-0.3455
	H/hn	0.2163
	C/c	0.6731
	O/o	-0.5426
	C6M/c3	-0.3985
	HM1/hc	0.1150
	HM2/hc	0.1150
	HM3/hc	0.1150
	S1/ss	-0.1387
	C2/cc	0.4438
	N3/nd	-0.5862
	N2/nh	-0.4939
	H2/hn	0.3287
	C2M/c3	-0.0110
	H3M/h1	0.0695
	H2M/h1	0.0695
	H1M/h1	0.0695

1E4	Atom name/type	RESP charge (*e*)	Atom name/type	RESP charge (*e*)
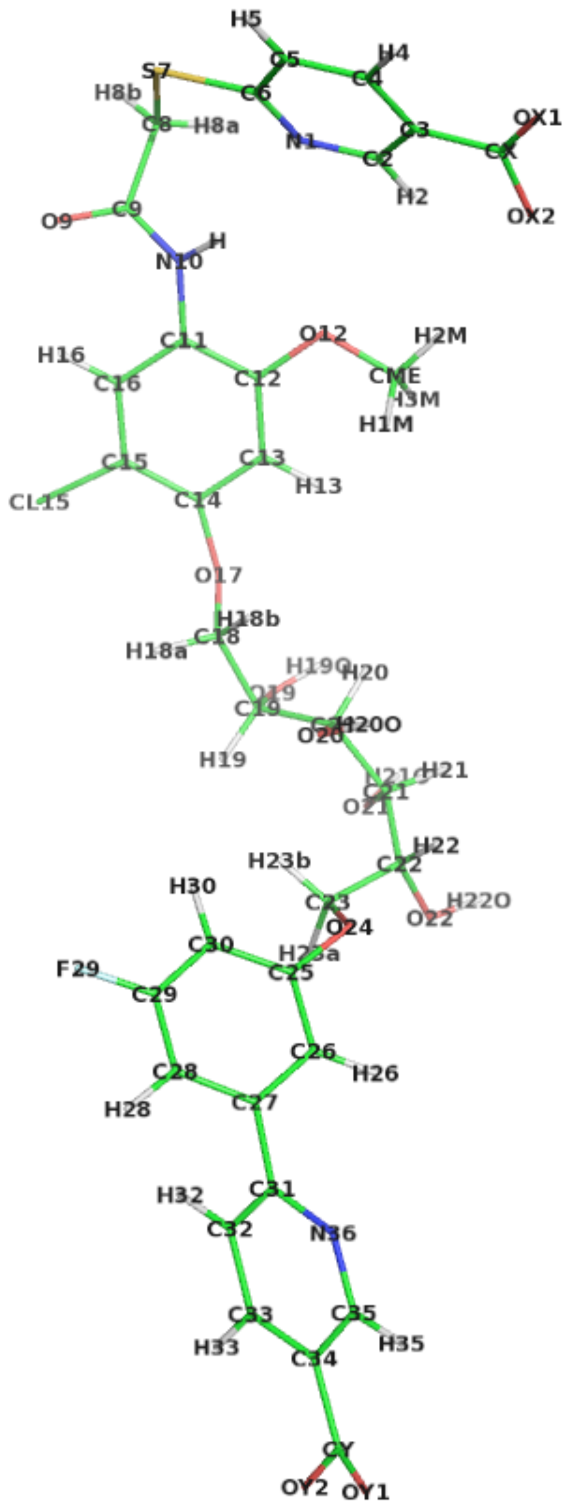	CL15/cl	-0.0957	C30/cp	-0.1622
	CME/c3	-0.0817	C31/ca	0.4005
	CX/c	0.7351	C32/ca	-0.3069
	OX1/o	-0.7607	C33/ca	-0.0887
	OX2/o	-0.7607	C34/ca	-0.0498
	N1/nb	-0.5190	C35/ca	0.1687
	C2/ca	0.1436	N36/nb	-0.5444
	C3/ca	-0.0214	CY/c	0.7581
	C4/ca	-0.1431	OY1/o	-0.7683
	C5/ca	-0.2715	OY2/o	-0.7683
	C6/ca	0.4507	F29/f	-0.1906
	S7/ss	-0.3197	H2/h4	0.0940
	C8/c3	-0.2161	H4/ha	0.1530
	C9/c	0.6093	H5/ha	0.1733
	O9/o	-0.5923	H10/hn	0.1828
	N10/n	-0.2360	H13/ha	0.1460
	C11/ca	-0.0080	H16/ha	0.1585
	C12/ca	0.0671	H19/h1	0.1105
	O12/os	-0.1875	H20/h1	0.0606
	C13/ca	-0.1585	H21/h1	0.0606
	C14/ca	0.1972	H22/h1	0.1105
	C15/ca	-0.0398	H26/ha	0.1523
	C16/ca	-0.0837	H28/ha	0.1267
	O17/os	-0.3504	H30/ha	0.1442
	C18/c3	0.0149	H32/ha	0.1285
	C19/c3	0.2806	H33/ha	0.1429
	O19/oh	-0.6489	H35/h4	0.0879
	C20/c3	0.0834	H1M/h1	0.0873
	O20/oh	-0.6900	H2M/h1	0.0873
	C21/c3	0.0834	H3M/h1	0.0873
	O21/oh	-0.6900	H8a/h1	0.1297
	C22/c3	0.2806	H8b/h1	0.1297
	O22/oh	-0.6489	H19O/ho	0.4178
	C23/c3	0.0149	H18A/h1	0.0673
	O24/os	-0.3642	H18B/h1	0.0673
	C25/ca	0.1291	H20O/ho	0.4504
	C26/ca	-0.1546	H21O/ho	0.4504
	C27/cp	-0.1198	H22O/ho	0.4178
	C28/ca	-0.0859	H23A/h1	0.0673
	C29/ca	0.1509	H23B/h1	0.0673

6P3	Atom name/type	RESP charge (*e*)
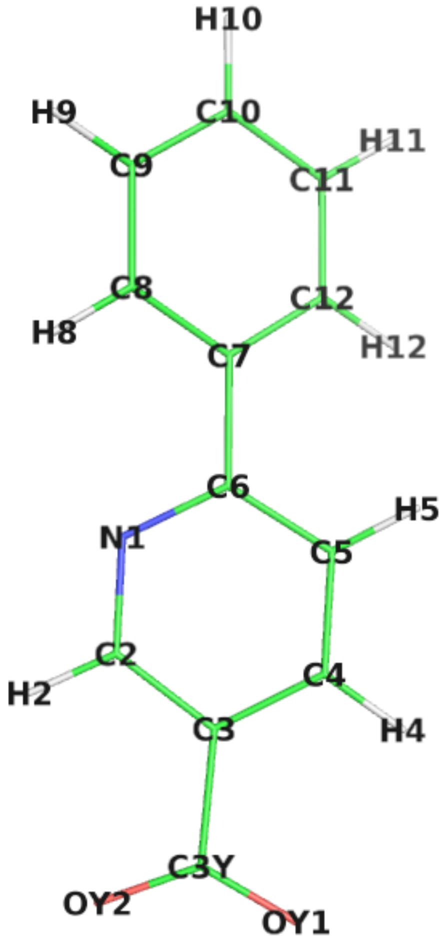	C11/ca	-0.1689
	H11/ha	0.1255
	C12/ca	-0.1021
	H12/ha	0.1107
	C7/cp	-0.0105
	C8/ca	-0.1021
	H8/ha	0.1107
	C9/ca	-0.1689
	H9/ha	0.1255
	C10/ca	-0.1440
	H10/ha	0.1192
	C6/cp	0.3521
	C5/ca	-0.3171
	H5/ha	0.1181
	C4/ca	-0.0625
	H4/ha	0.1324
	C3/ca	-0.0516
	C2/ca	0.1581
	H2/h4	0.0805
	N1/nb	-0.5167
	C3Y/c	0.7426
	OY1/o	-0.7655
	OY2/o	-0.7655

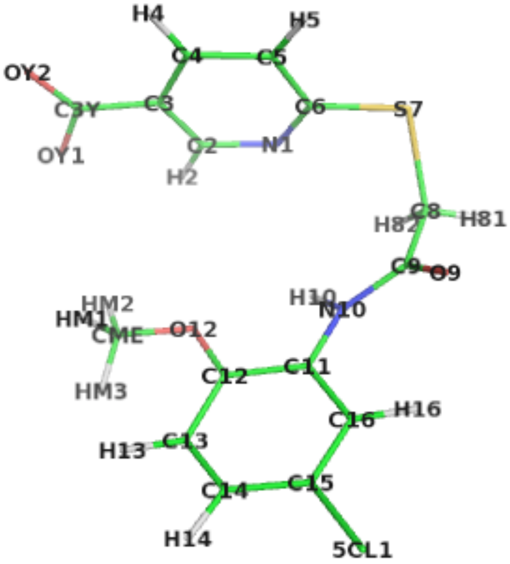

**1E7**	**Atom name/type**	**RESP charge (*e*)**
	C2/ca	0.1382
	H2/h4	0.0939
	C3/ca	-0.0201
	C4/ca	-0.1496
	H4/ha	0.1525
	C5/ca	-0.2493
	H5/ha	0.1688
	C6/ca	0.4212
	N1/nb	-0.5055
	S7/ss	-0.3175
	C8/c3	-0.2038
	H81/h1	0.1276
	H82/h1	0.1276
	C9/c	0.5966
	O9/o	-0.5891
	C3Y/c	0.7437
	OY1/o	-0.7634
	OY2/o	-0.7634
	C11/ca	-0.0196
	C12/ca	0.1457
	C13/ca	-0.1709
	H13/ha	0.1399
	C14/ca	-0.1294
	H14/ha	0.1554
	C15/ca	-0.0611
	CL15/cl	-0.1155
	C16/ca	-0.0133
	H16/ha	0.1515
	O12/os	-0.2279
	CME/c3	-0.0702
	HM1/h1	0.0852
	HM2/h1	0.0852
	HM3/h1	0.0852
	N10/n	-0.2426
	H10/hn	0.1940

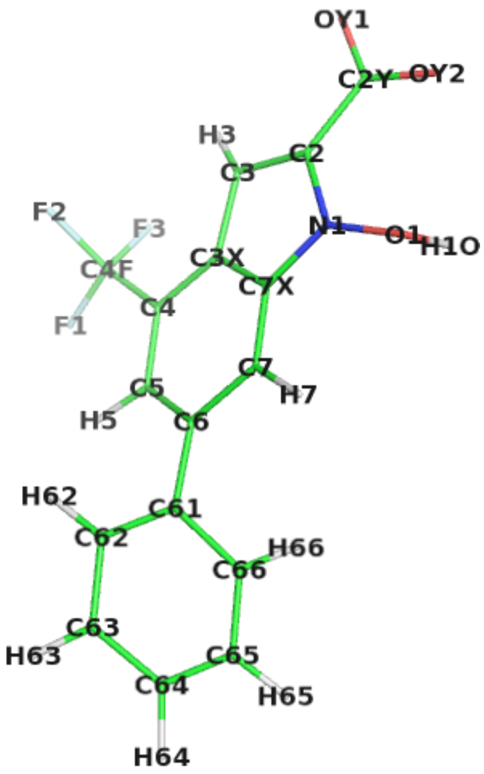

NHI	Atom name/type	RESP charge (*e*)
	C3X/ca	0.0256
	C7X/ca	0.0647
	C7/ca	-0.1397
	H7/ha	0.1339
	C6/cp	-0.0943
	C5/ca	-0.1500
	H5/ha	0.1168
	C4/ca	-0.0404
	C4F/c3	0.4717
	F1/f	-0.1845
	F2/f	-0.1845
	F3/f	-0.1845
	C61/cp	0.0531
	C62/ca	-0.0828
	H62/ha	0.1147
	C63/ca	-0.2059
	H63/ha	0.1396
	C64/ca	-0.1165
	H64/ha	0.1231
	C65/ca	-0.2059
	H65/ha	0.1396
	C66/ca	-0.0828
	H66/ha	0.1147
	N1/na	0.1382
	C2/cc	0.0260
	C3/cd	-0.4338
	H3/ha	0.2198
	C2Y/c	0.8632
	OY1/o	-0.7751
	OY2/o	-0.7751
	O1/oh	-0.5359
	H1O/ho	0.4470

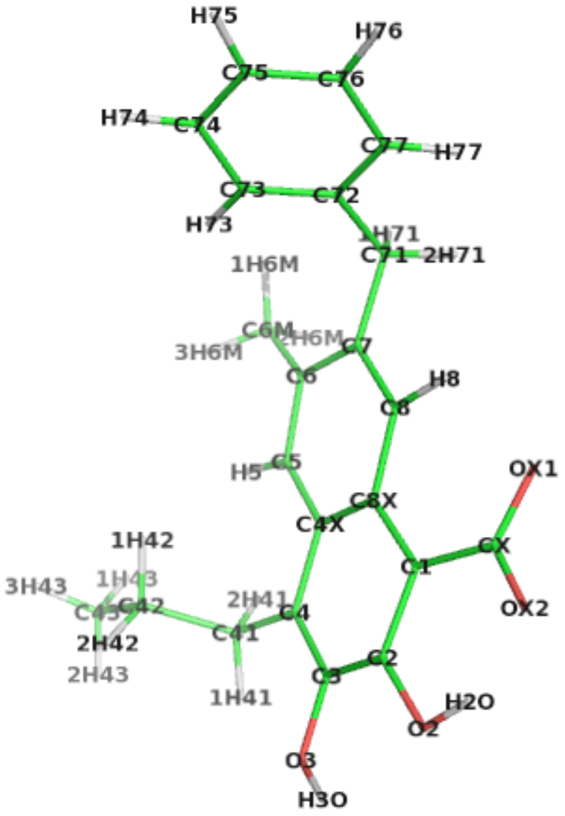

FX11	Atom name/type	RESP charge (*e*)	Atom name/type	RESP charge (*e*)
	C8X/ca	-0.1178	C5/ca	-0.2087
	C4/ca	-0.1468	H5/ha	0.1672
	C4X/ca	-0.0574	C73/ca	-0.1191
	C7/ca	0.0568	H73/ha	0.1197
	C72/ca	0.1110	C74/ca	-0.2067
	C6/ca	0.0380	H74/ha	0.1418
	C1/ca	-0.0387	C75/ca	-0.1189
	C3/ca	0.2742	H75/ha	0.1197
	C2/ca	0.1397	C76/ca	-0.2067
	CX/c	0.7865	H76/ha	0.1418
	H2O/ho	0.4133	C77/ca	-0.1191
	H3O/ho	0.4113	H77/ha	0.1197
	O2/oh	-0.5781		
	O3/oh	-0.6103		
	OX1/o	-0.7659		
	OX2/o	-0.7659		
	C43/c3	-0.0994		
	1H43/hc	0.0121		
	2H43/hc	0.0121		
	3H43/hc	0.0121		
	C42/c3	0.0106		
	1H42/hc	0.0180		
	2H42/hc	0.0180		
	C41/c3	0.0259		
	1H41/hc	0.0201		
	2H41/hc	0.0201		
	C6M/c3	-0.2712		
	1H6M/hc	0.0680		
	2H6M/hc	0.0680		
	3H6M/hc	0.0680		
	C71/c3	-0.0974		
	2H71/hc	0.0285		
	2H72/hc	0.0285		
	C8/ca	-0.1215		
	H8/ha	0.1989		
