# Supplementary figures and images for: Human Lactate Dehydrogenase A Inhibitors: A Molecular Dynamics Investigation
Source: PLoS One. 2014 Jan 17;9(1):e86365. doi: 10.1371/journal.pone.0086365 (PMC3895040; doi:10.1371/journal.pone.0086365)

## Text S2. Root mean squared deviation (RMSD) of LDHA backbone atoms.

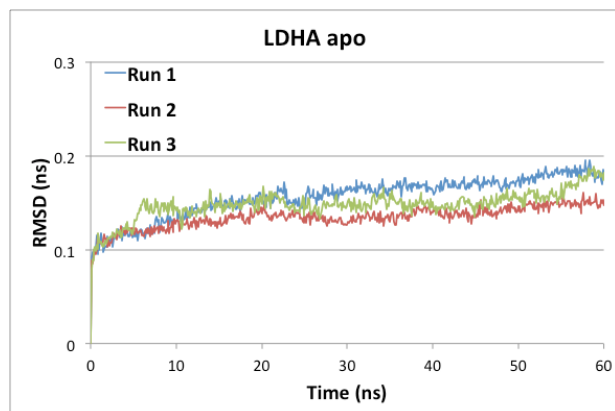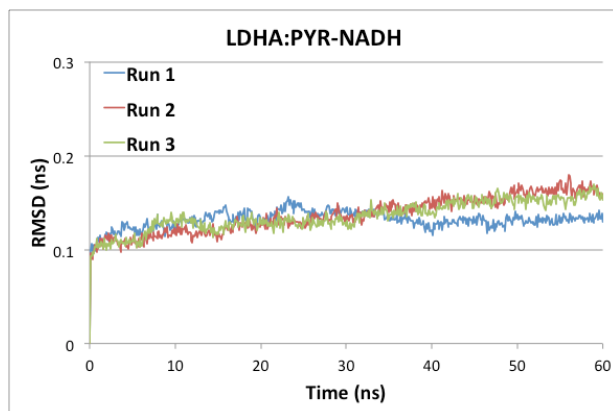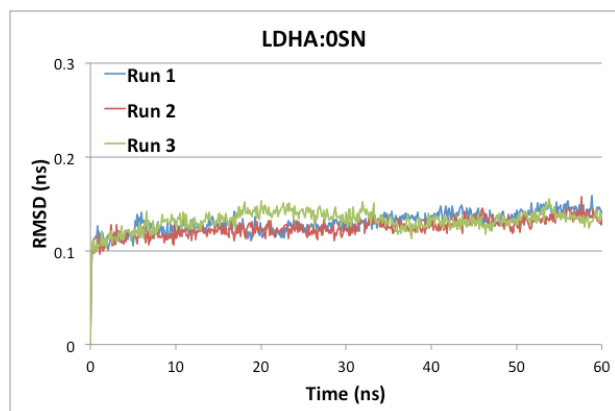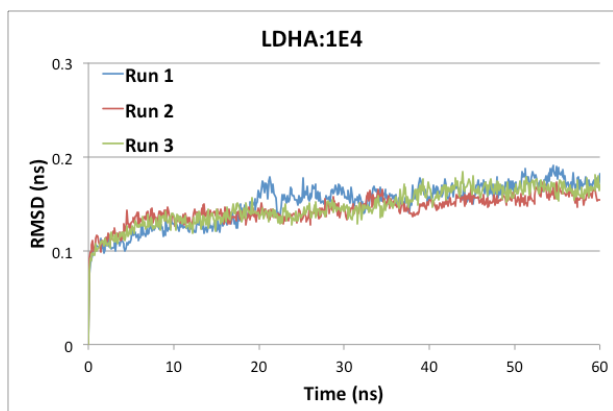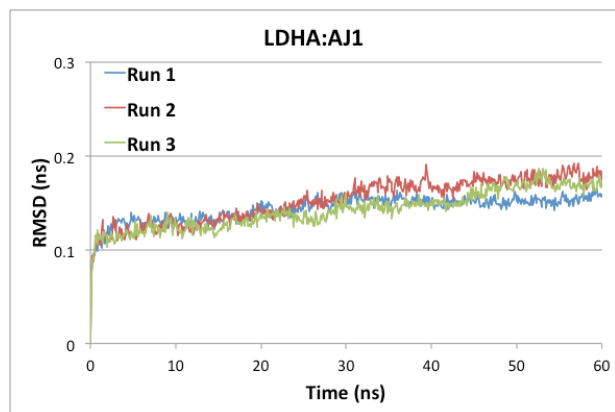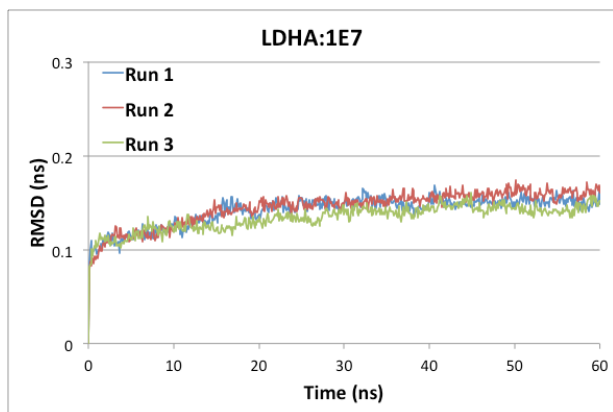

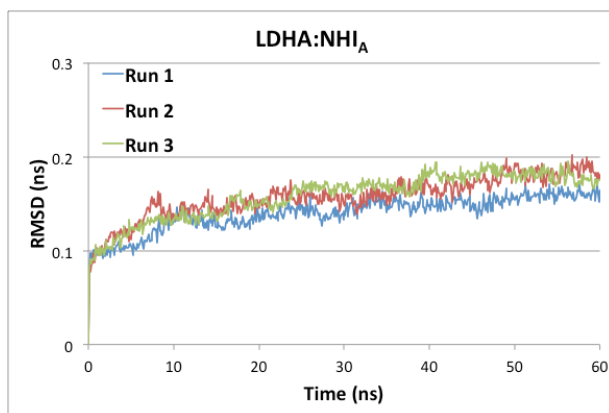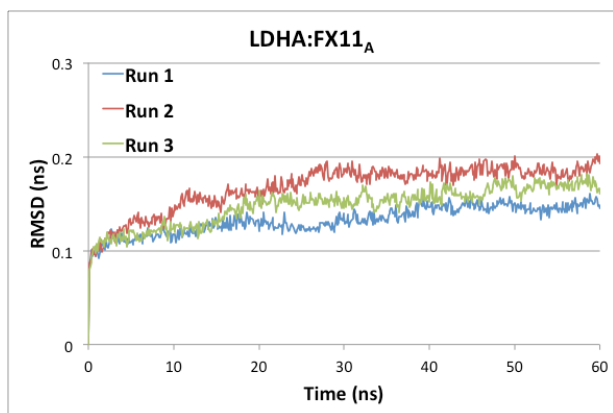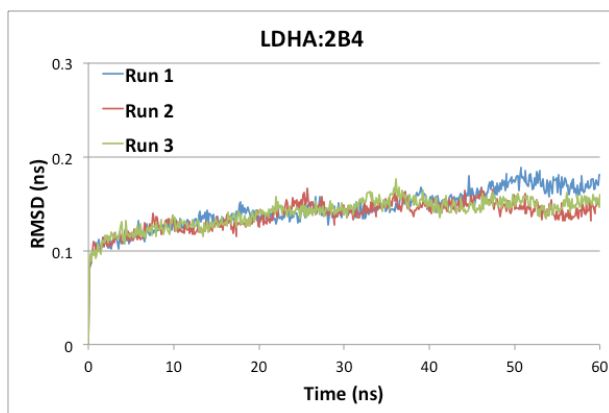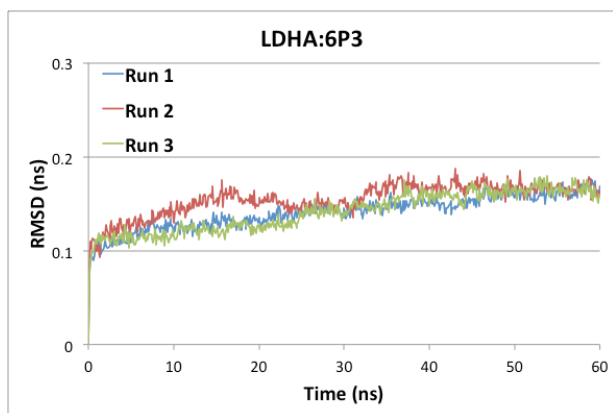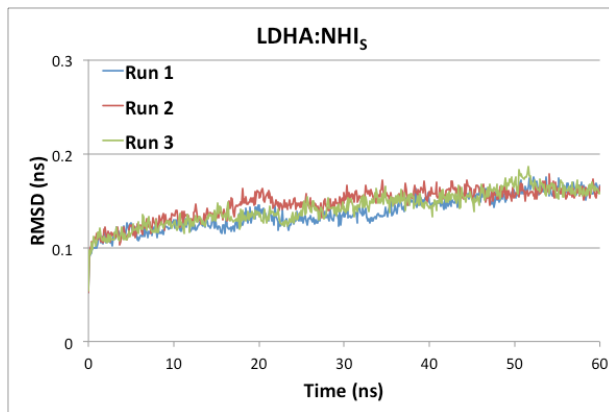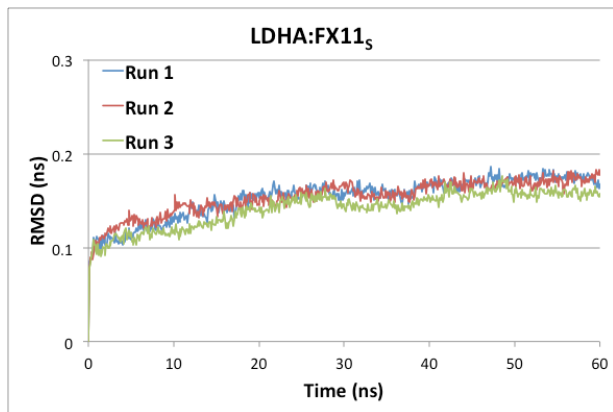

Supplement: Text S2 — Root mean squared deviation (RMSD) of LDHA backbone atoms. (PDF) [file pone.0086365.s006.pdf]
