# Supplementary material for: Human Lactate Dehydrogenase A Inhibitors: A Molecular Dynamics Investigation
Source: PLoS One. 2014 Jan 17;9(1):e86365. doi: 10.1371/journal.pone.0086365 (PMC3895040; doi:10.1371/journal.pone.0086365)
Supplement: Text S3 — Root mean squared deviation (RMSD) of heavy atoms of selected binding site residues and ligands. (PDF) [file pone.0086365.s007.pdf]

Text S3. Root mean squared deviation (RMSD) of heavy atoms of selected binding site residues and ligands.

The following residues, within 0.5 nm of 0SN, were selected based on the human LDHA crystal structure 4AJP: Val25, Gly26, Val27, Gly28, Ala29, Val30, Gly31, Val50, Asp51, Val52, Tyr82, Thr94, Ala95, Gly96, Ala97, Arg98, Gln99, Gln100, Glu101, Gly102, Glu103, Ser104, Arg105, Ile115, Phe118, Ile119, Val135, Ser136, Asn137, Leu164, Arg168, His192, Ala237, Thr247, Ile251.

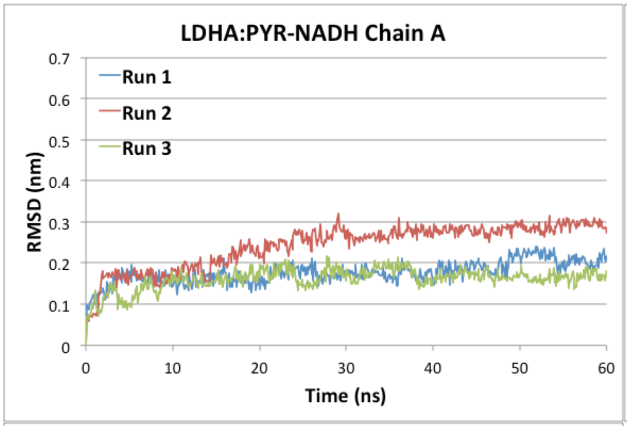

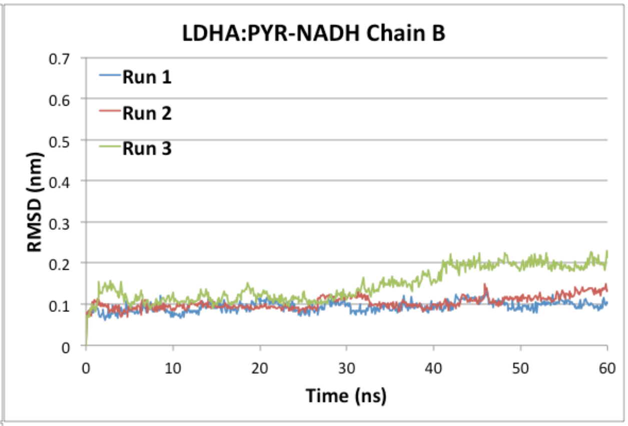

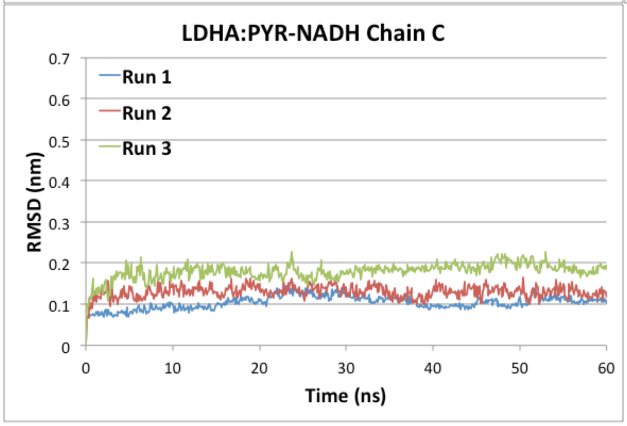

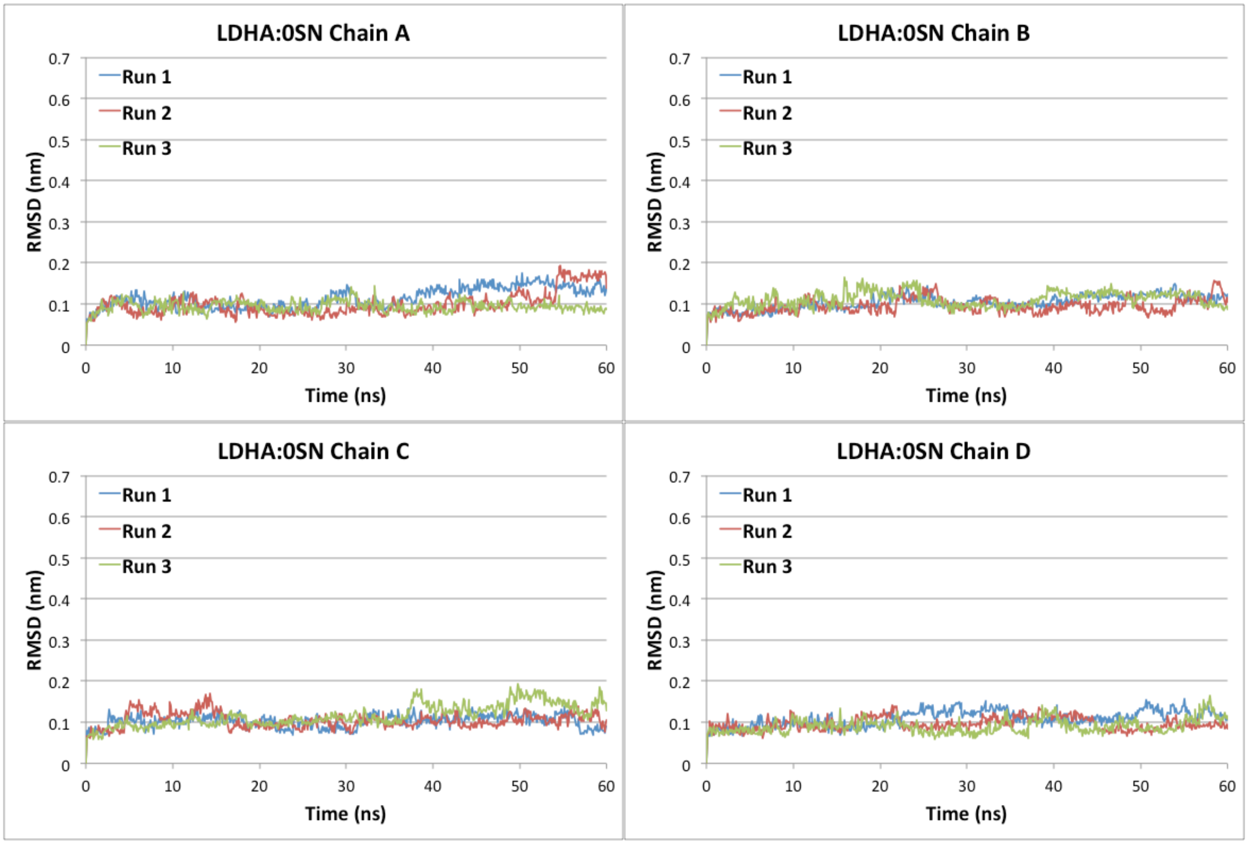

LDHA:0SN Chain A
LDHA:0SN Chain B
LDHA:0SN Chain C
LDHA:0SN Chain D
Run 1
Run 2
Run 3
RMSD (nm)
Time (ns)
0
0.1
0.2
0.3
0.4
0.5
0.6
0.7
0
10
20
30
40
50
60

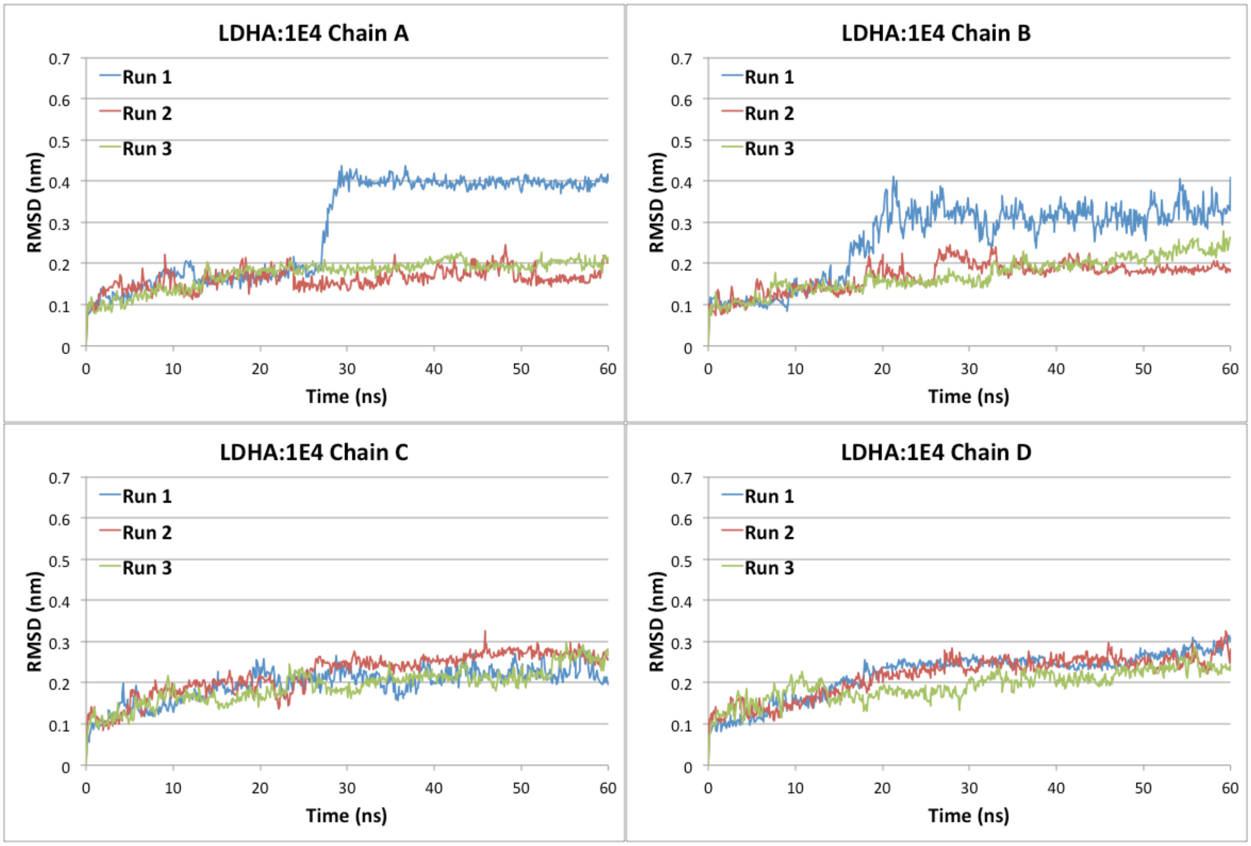

LDHA:1E4 Chain A
LDHA:1E4 Chain B
LDHA:1E4 Chain C
LDHA:1E4 Chain D
Run 1
Run 2
Run 3
RMSD (nm)
Time (ns)
0
0.1
0.2
0.3
0.4
0.5
0.6
0.7
0
10
20
30
40
50
60

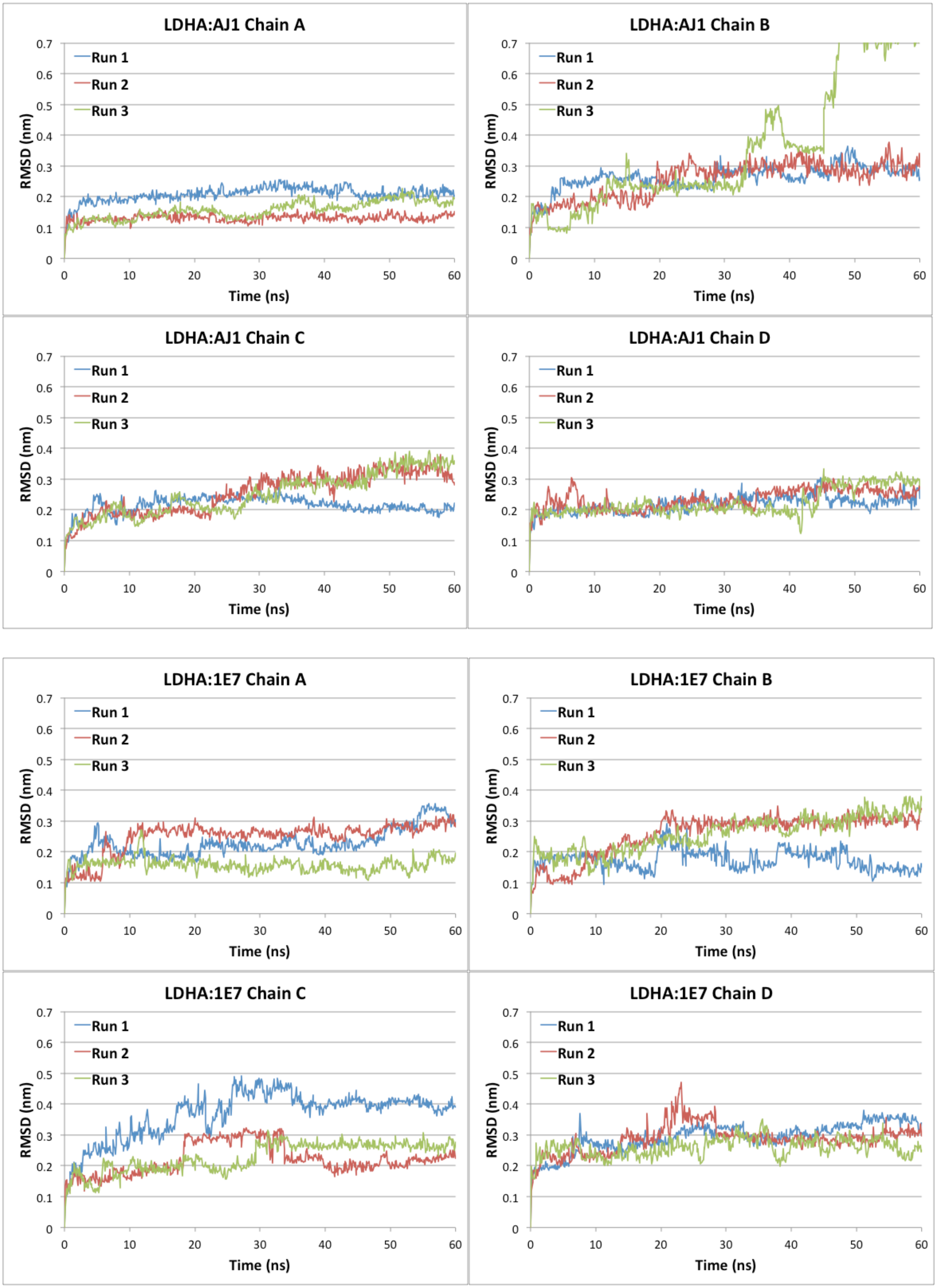
LDHA:AJ1 Chain A
LDHA:AJ1 Chain B
LDHA:AJ1 Chain C
LDHA:AJ1 Chain D
LDHA:1E7 Chain A
LDHA:1E7 Chain B
LDHA:1E7 Chain C
LDHA:1E7 Chain D
Run 1
Run 2
Run 3
RMSD (nm)
Time (ns)
0
0.1
0.2
0.3
0.4
0.5
0.6
0.7
10
20
30
40
50
60

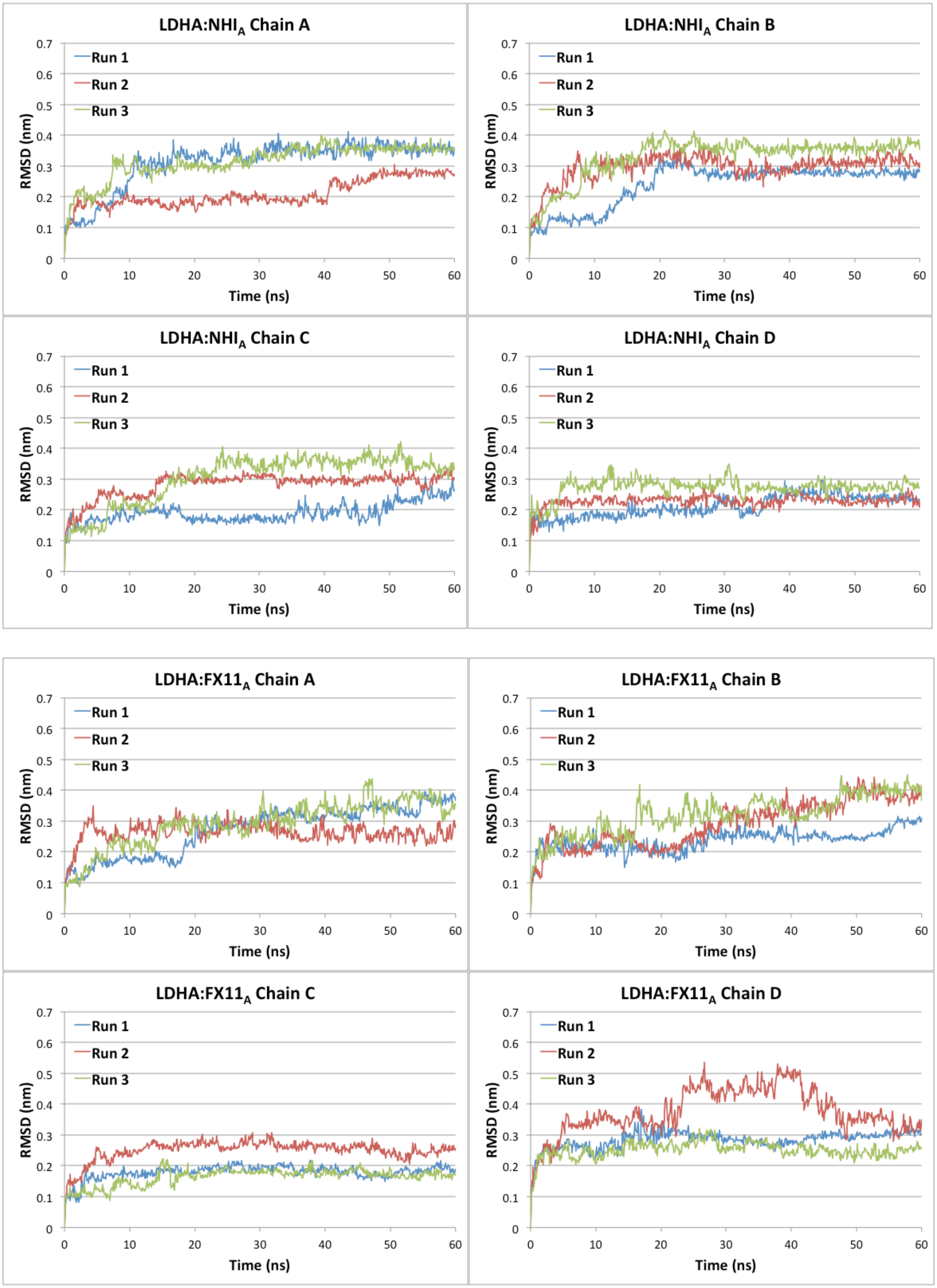

LDHA:NHI$_A$ Chain A
LDHA:NHI$_A$ Chain B
LDHA:NHI$_A$ Chain C
LDHA:NHI$_A$ Chain D
LDHA:FX11$_A$ Chain A
LDHA:FX11$_A$ Chain B
LDHA:FX11$_A$ Chain C
LDHA:FX11$_A$ Chain D
Run 1
Run 2
Run 3
RMSD (nm)
Time (ns)
0
0.1
0.2
0.3
0.4
0.5
0.6
0.7
10
20
30
40
50
60

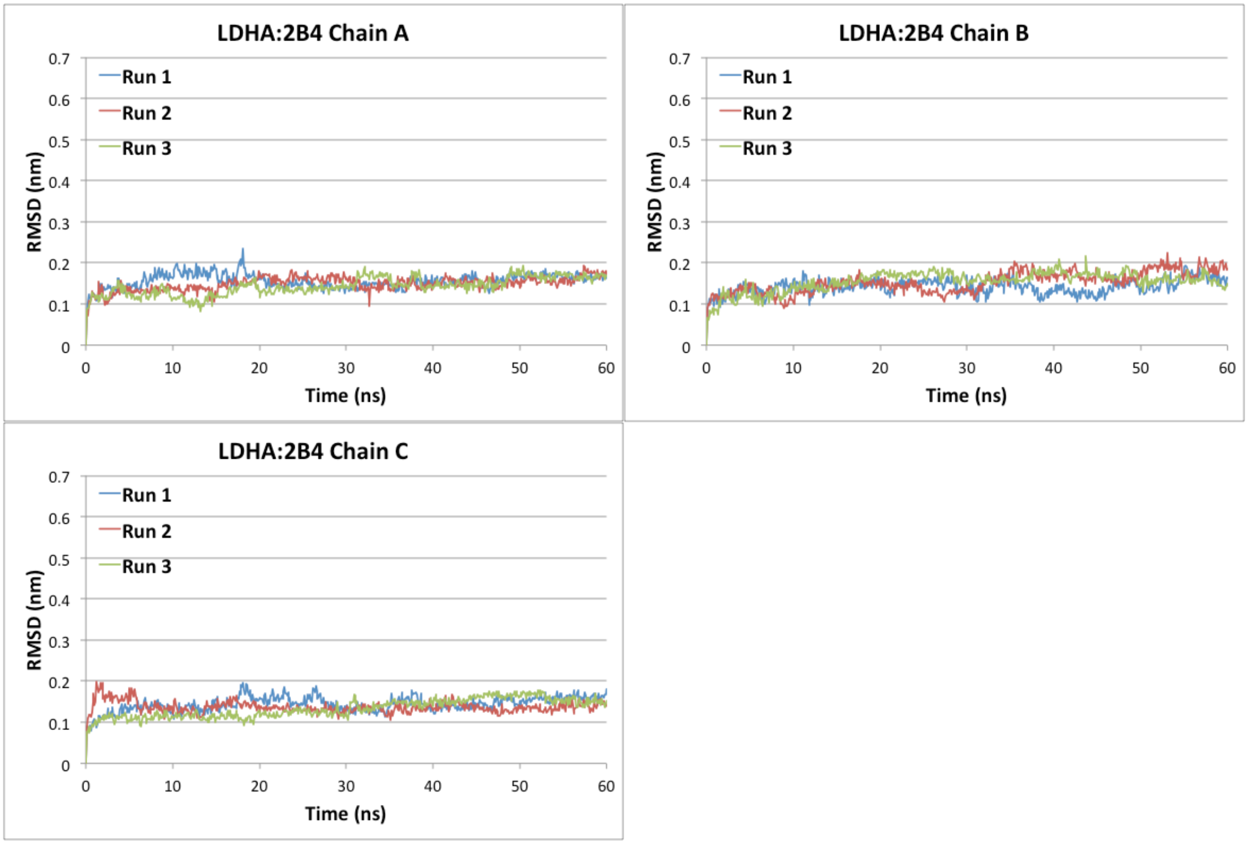
LDHA:2B4 Chain A
LDHA:2B4 Chain B
LDHA:2B4 Chain C
RMSD (nm)
Time (ns)
Run 1
Run 2
Run 3
0
0.1
0.2
0.3
0.4
0.5
0.6
0.7
10
20
30
40
50
60

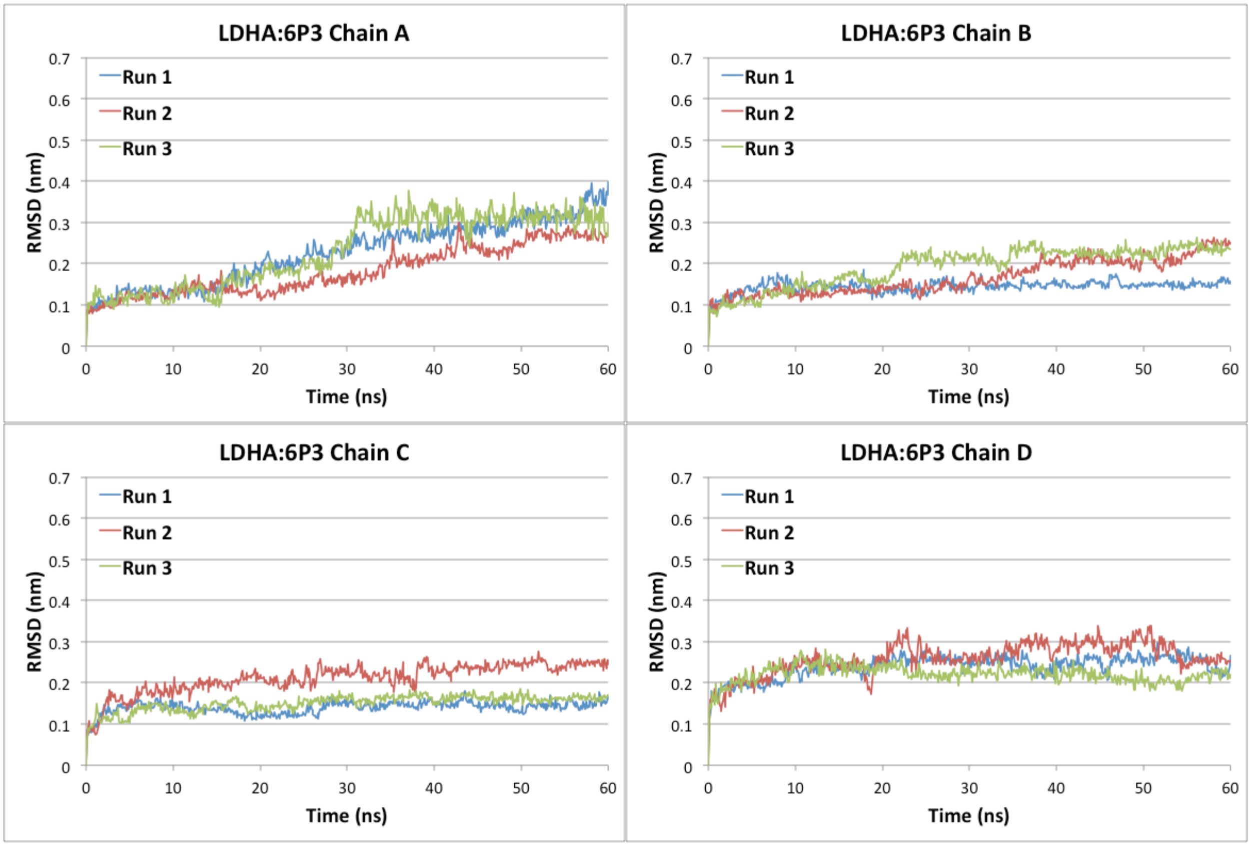
LDHA:6P3 Chain A
LDHA:6P3 Chain B
LDHA:6P3 Chain C
LDHA:6P3 Chain D
RMSD (nm)
Time (ns)
Run 1
Run 2
Run 3
0
0.1
0.2
0.3
0.4
0.5
0.6
0.7
10
20
30
40
50
60

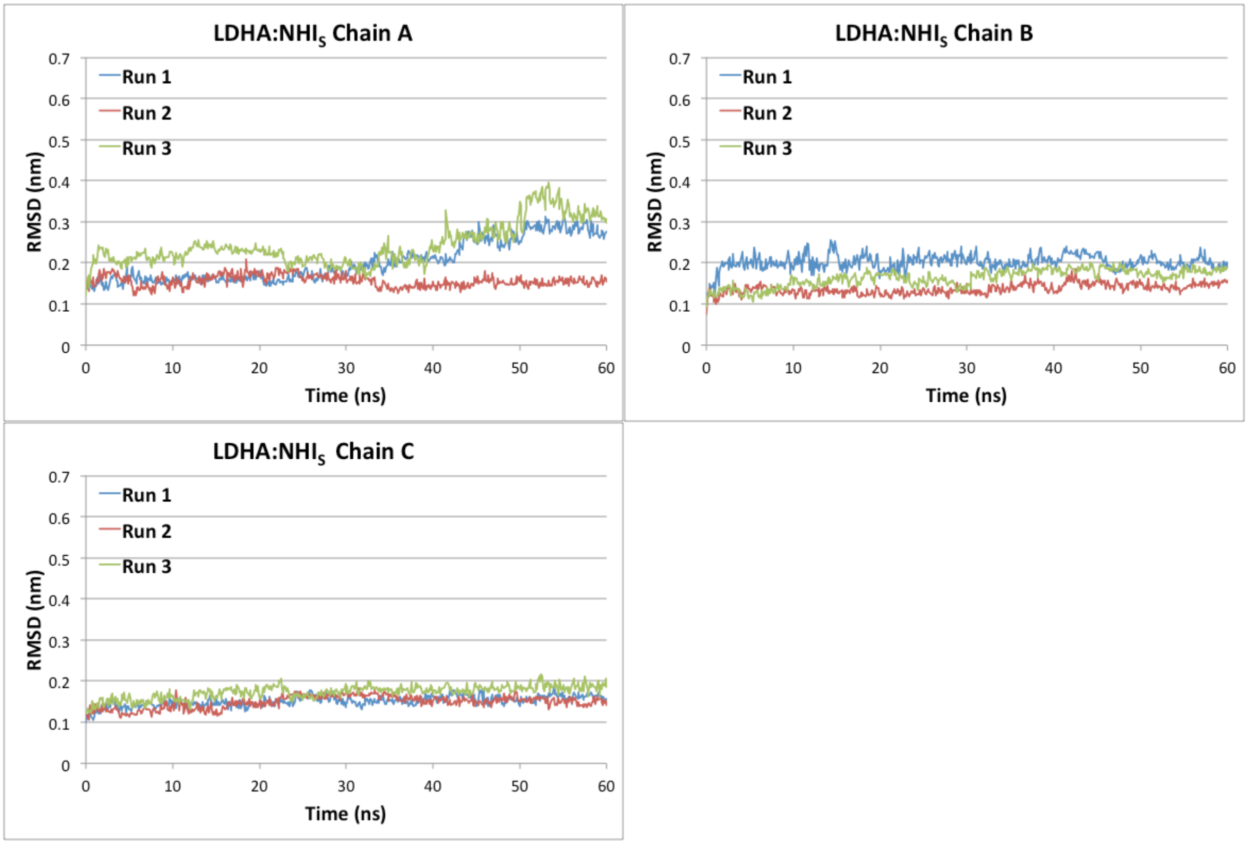
LDHA:NHI$_S$ Chain A
LDHA:NHI$_S$ Chain B
LDHA:NHI$_S$ Chain C
Run 1
Run 2
Run 3
RMSD (nm)
Time (ns)
0
0.1
0.2
0.3
0.4
0.5
0.6
0.7
10
20
30
40
50
60

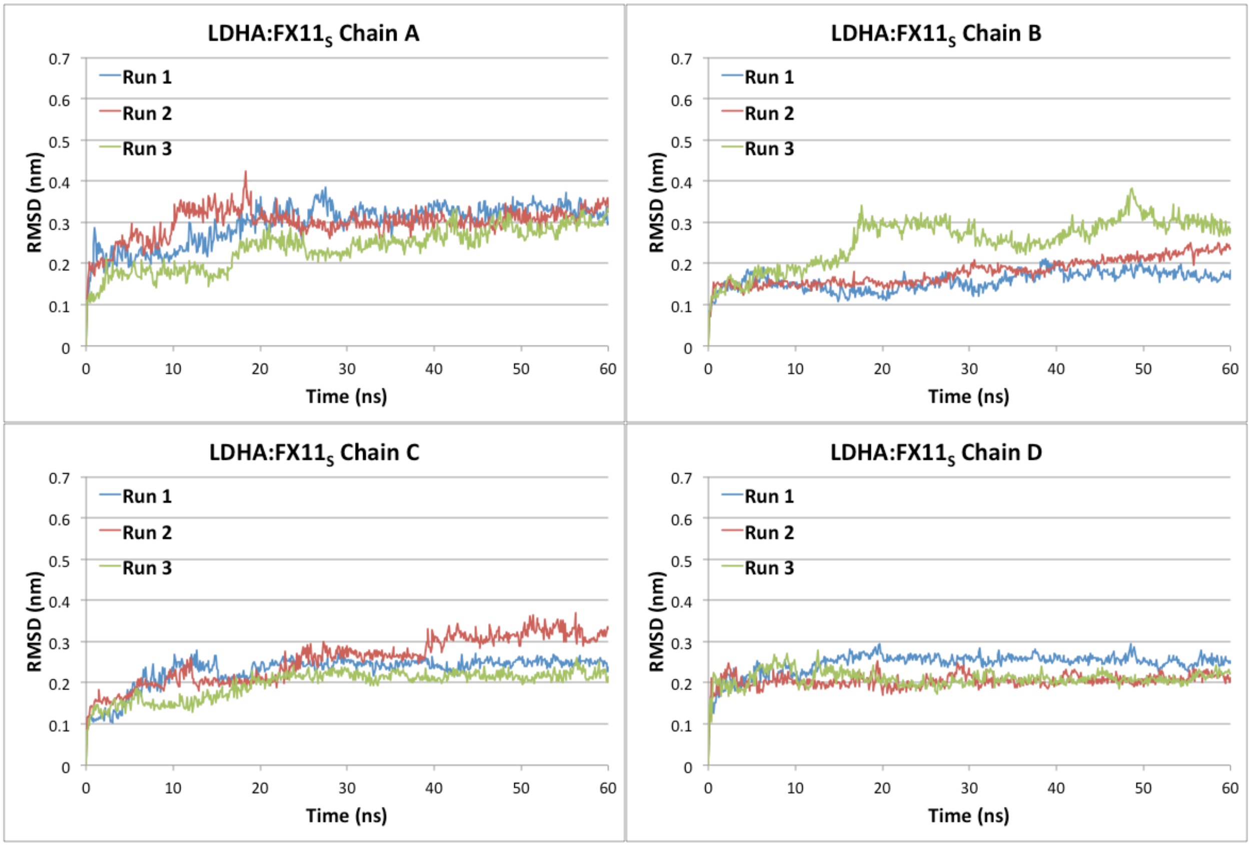
LDHA:FX11$_S$ Chain A
LDHA:FX11$_S$ Chain B
LDHA:FX11$_S$ Chain C
LDHA:FX11$_S$ Chain D
Run 1
Run 2
Run 3
RMSD (nm)
Time (ns)
0
0.1
0.2
0.3
0.4
0.5
0.6
0.7
10
20
30
40
50
60
