# Supplementary material for: Human Lactate Dehydrogenase A Inhibitors: A Molecular Dynamics Investigation
Source: PLoS One. 2014 Jan 17;9(1):e86365. doi: 10.1371/journal.pone.0086365 (PMC3895040; doi:10.1371/journal.pone.0086365)
Supplement: Text S4 — Superimposition of cluster centroids. (PDF) [file pone.0086365.s008.pdf]

Text S4. Superimposition of cluster centroids.

Initial structures of binding sites, from either crystal structures or docking structures, were also overlaid for comparison. The carbon atom and cartoon are colored in green (chain A), cyan (chain B), magenta (chain C), yellow (chain D), and grey (initial structure). Ligands are shown in thick sticks while selected binding site residues are shown in thin lines. Other atoms are colored: oxygen, red; nitrogen, blue; phosphate, orange; sulfur, yellow; chlorine, green.

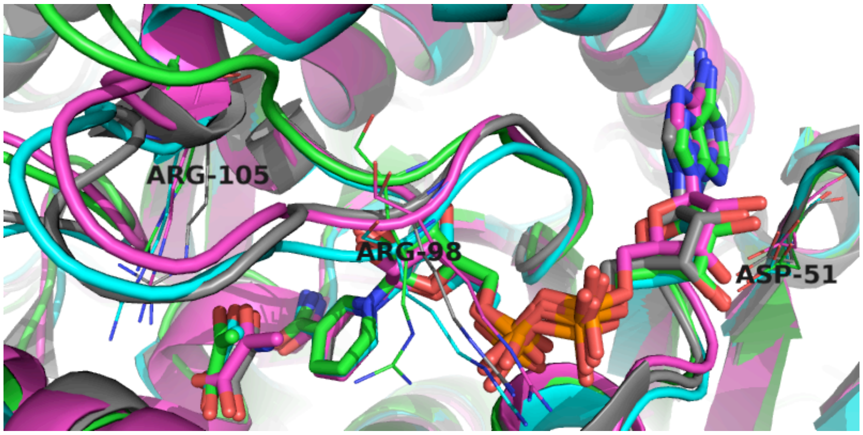

LDHA:PYR-NADH

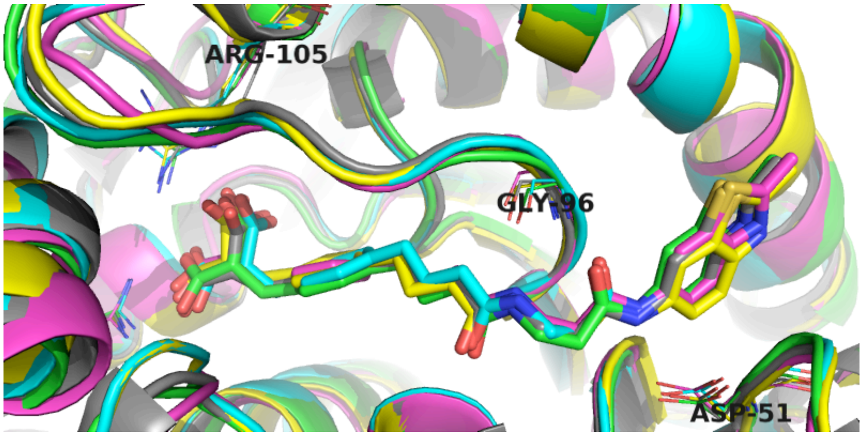

LDHA:0SN

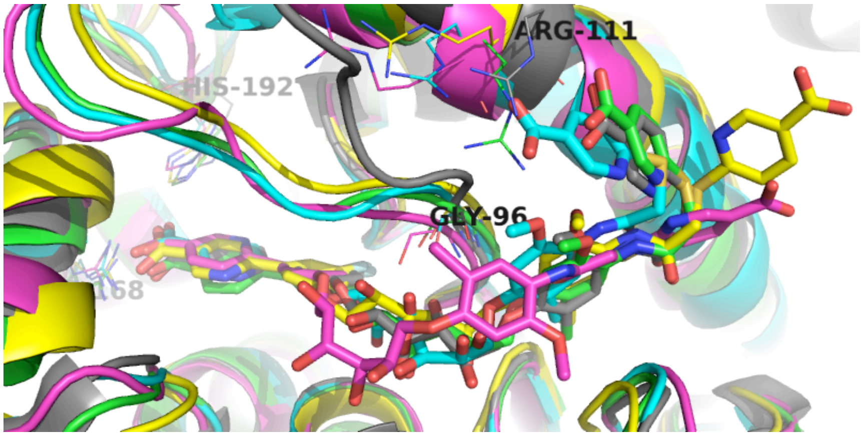

LDHA:1E4

LDHA:AJ1

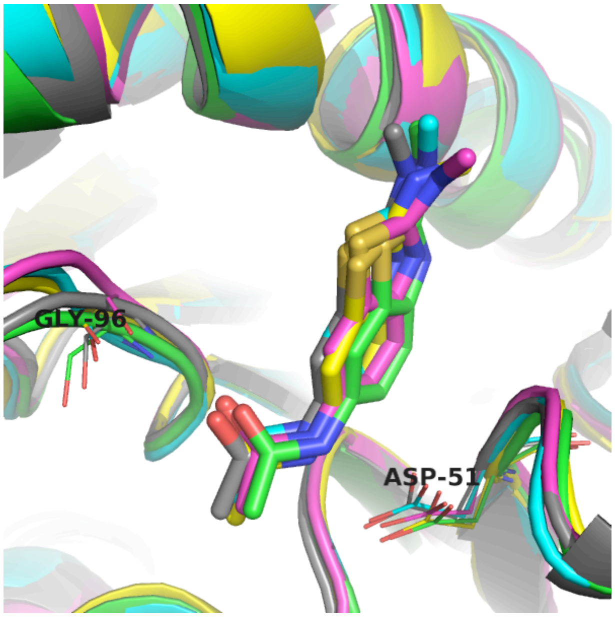

LDHA:1E7

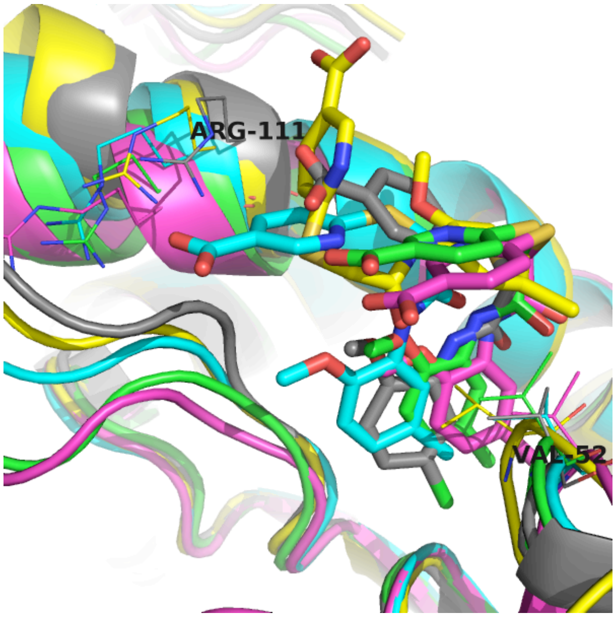

LDHA:NHI$_A$

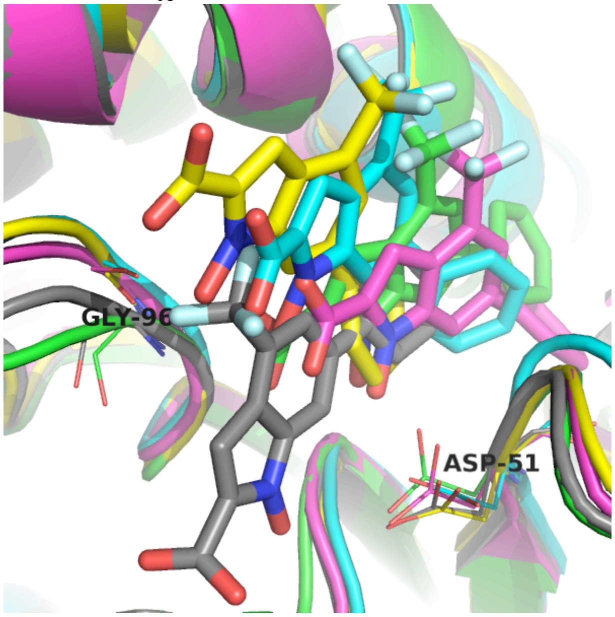

LDHA:FX11$_A$

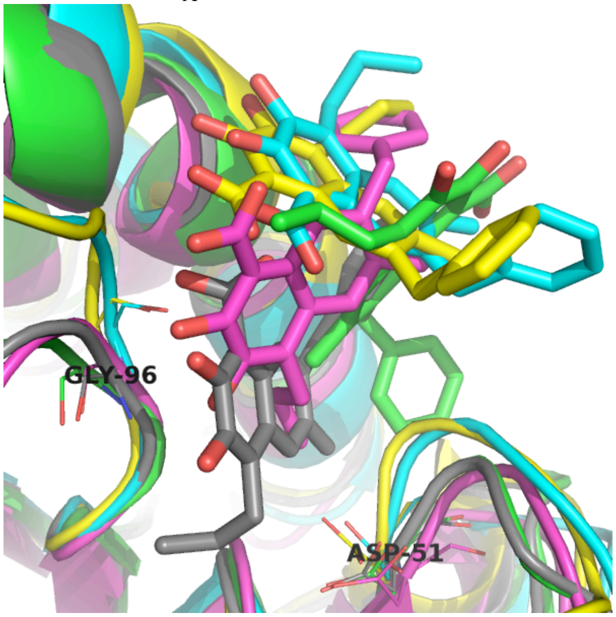

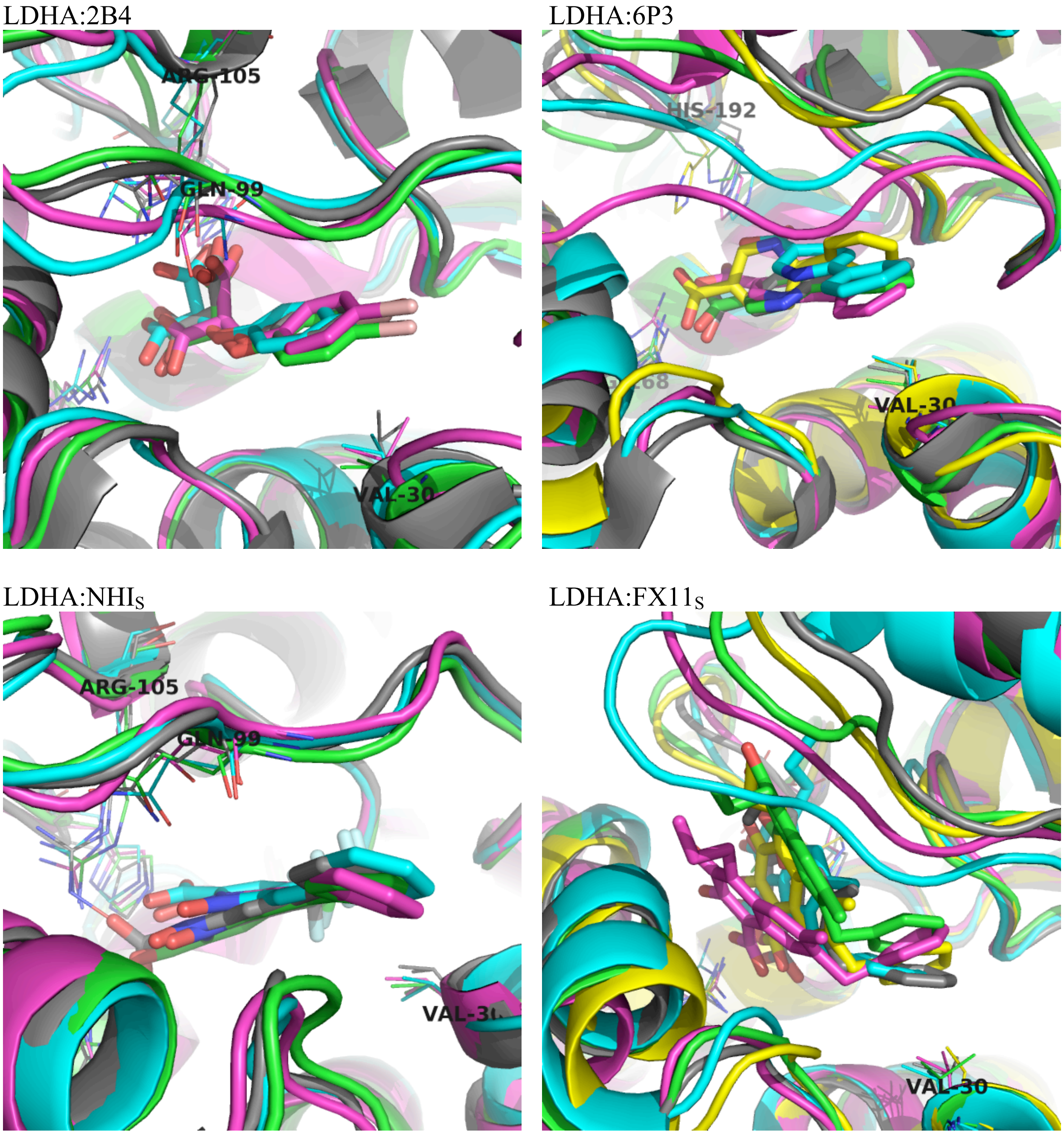
LDHA:2B4
ARG-105
GLN-99
VAL-30
LDHA:6P3
HIS-192
VAL-30
LDHA:NHI$_S$
ARG-105
GLN-99
LDHA:FX11$_S$
VAL-30
