# Supplementary material for: Human Lactate Dehydrogenase A Inhibitors: A Molecular Dynamics Investigation
Source: PLoS One. 2014 Jan 17;9(1):e86365. doi: 10.1371/journal.pone.0086365 (PMC3895040; doi:10.1371/journal.pone.0086365)
Supplement: Text S5 — Initial structures for steered MD simulations. (PDF) [file pone.0086365.s009.pdf]

Text S5. Initial structures for steered MD simulations.

The inhibitor was pulled towards you (the viewer). LDHA structure is labeled by green cartoon (backbone) and grey surface, whereas inhibitors are represented by thick sticks with atoms colored as: carbon, magenta; oxygen, red; nitrogen, blue; phosphate, orange; sulfur, yellow; chlorine, green; fluorine, pale cyan. The mobile loop is also colored in magenta for S-site binders.

AJ1

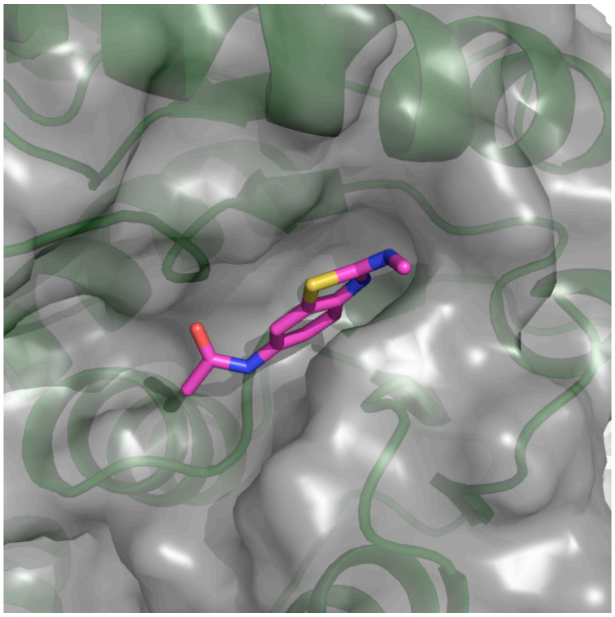

1E7

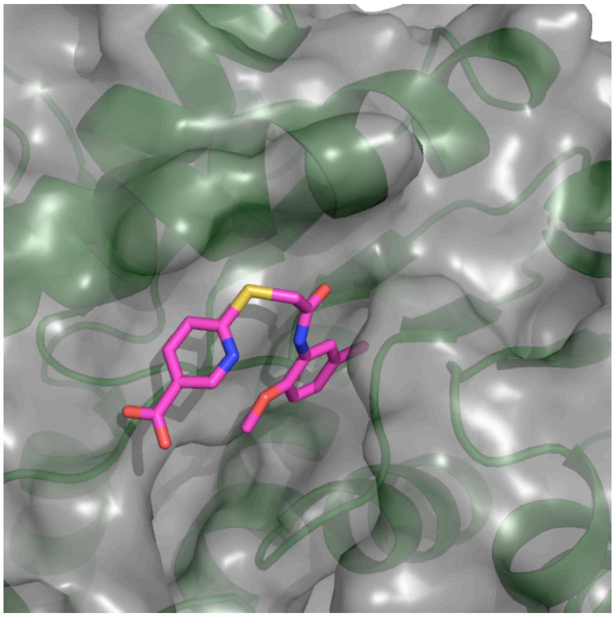

NHI_A

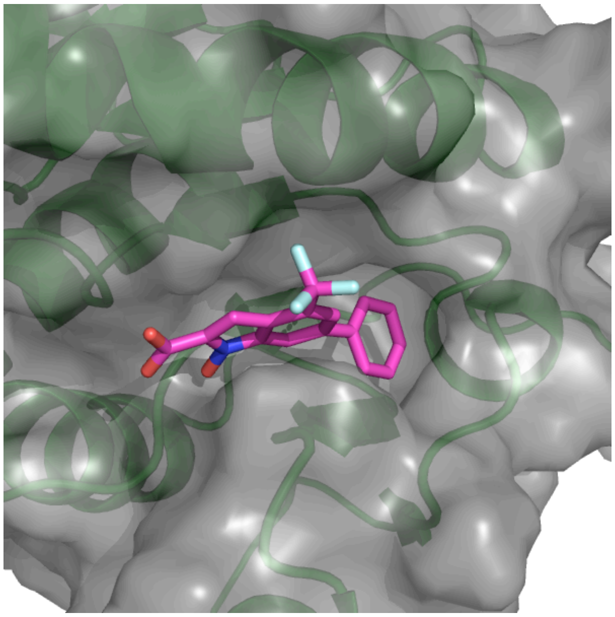

$FX11_A$

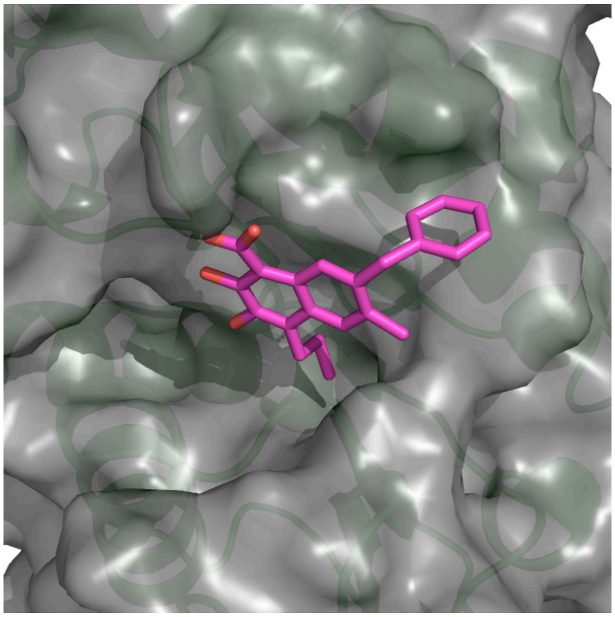

6P3 (loop open)

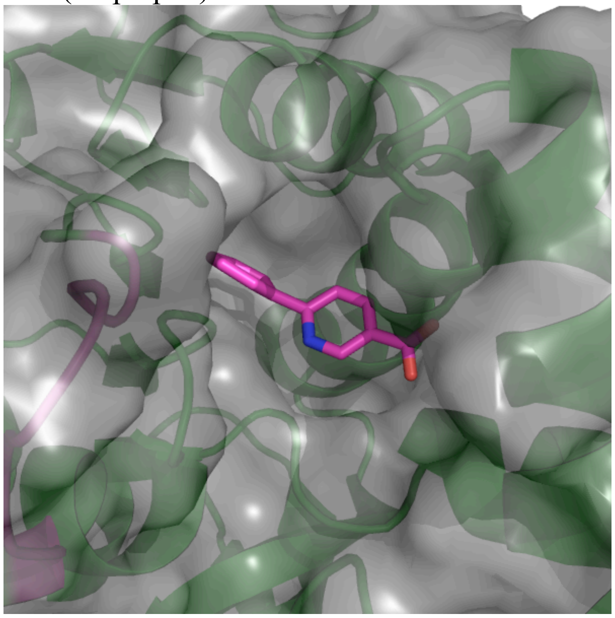

6P3 (loop closed)

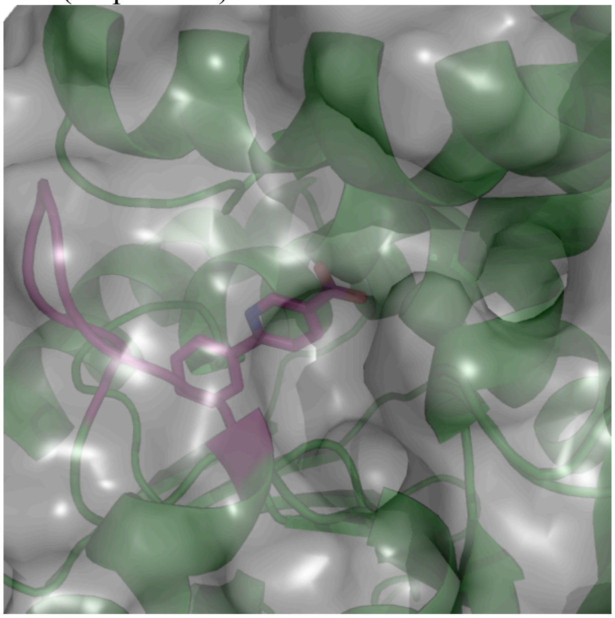

2B4 (A)

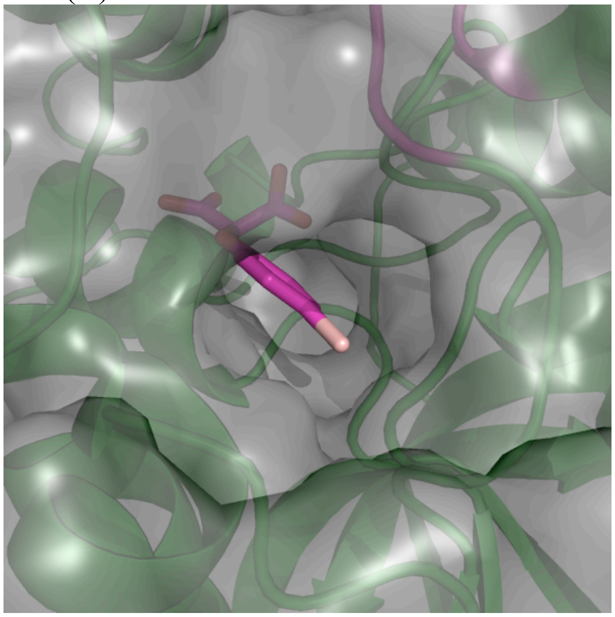

2B4 (B)

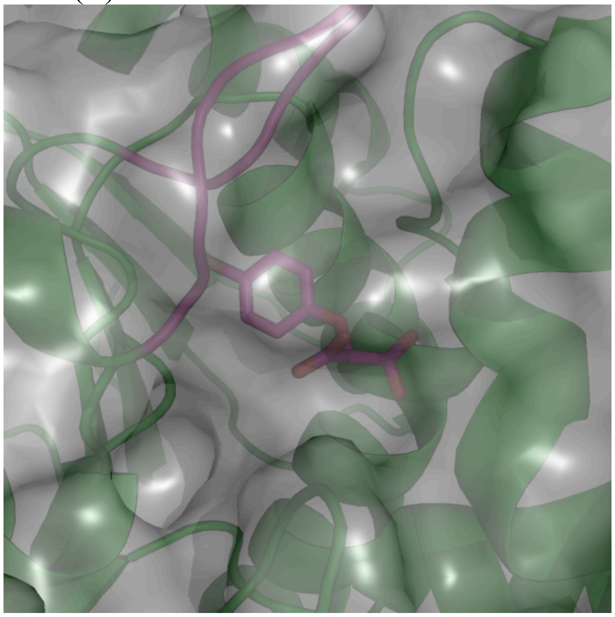

NHI_S

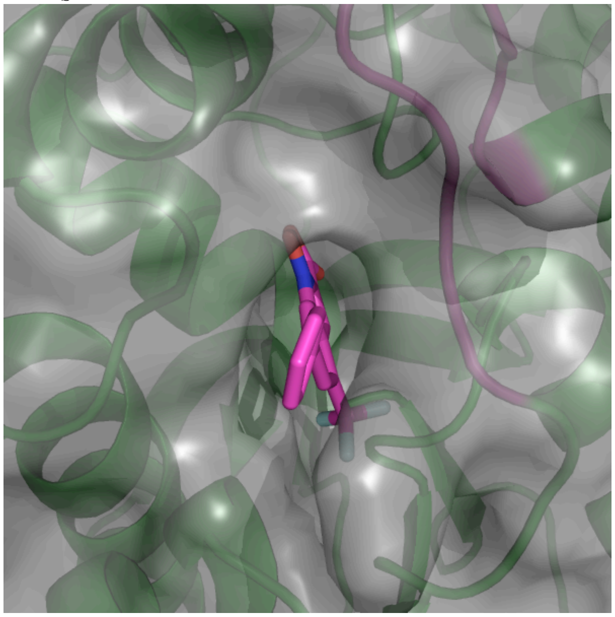

$FX11_S$

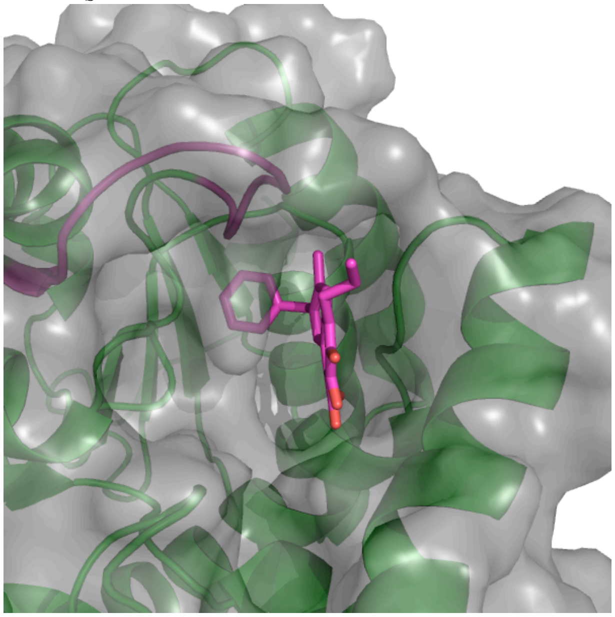

0SN

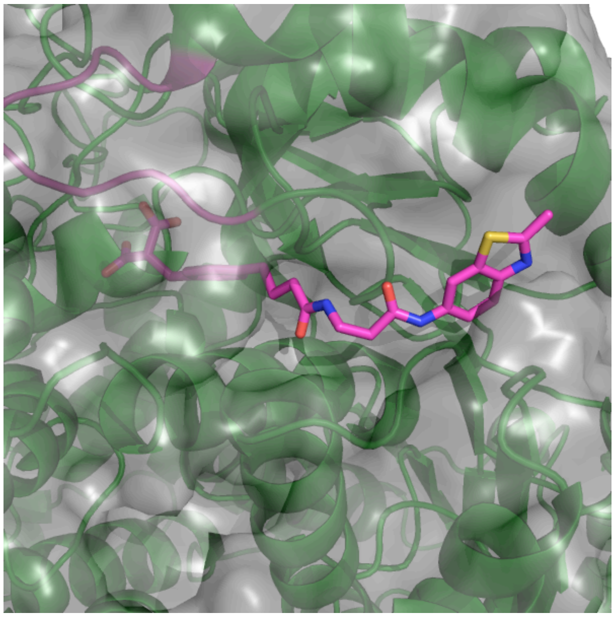

1E4

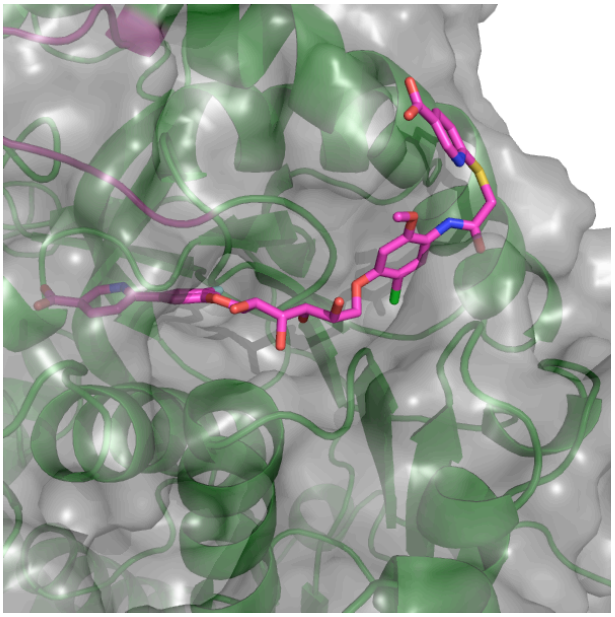

Apparently, the pulling of 6P3 (loop closed), 2B4 (A and B), and NHI_S, $FX11_S$, 0SN, and 1E4 is complicated by steric clashes of different extents.
