# Supplementary material for: Human Lactate Dehydrogenase A Inhibitors: A Molecular Dynamics Investigation
Source: PLoS One. 2014 Jan 17;9(1):e86365. doi: 10.1371/journal.pone.0086365 (PMC3895040; doi:10.1371/journal.pone.0086365)
Supplement: Text S6 — Original pulling work and peak force for steered MD runs. (PDF) [file pone.0086365.s010.pdf]

Text S6. Original pulling work and peak force for steered MD runs.

A-site binders	AJ1		1E7		NHI		FX11	
	W[a]	F[b]	W[a]	F[b]	W[a]	F[b]	W[a]	F[b]
Run 1	88.83	386.8	90.56	331.2	147.9	385.5	137.2	439.3
Run 2	137.9	302.8	103.5	335.3	149.6	419.7	131.5	380.4
Run 3	72.28	353.8	100.2	399.9	134.0	416.7	113.4	420.1
Run 4	108.9	352.9	84.50	324.4	136.8	455.2	132.1	408.6
Run 5	89.60	375.0	91.21	369.0	112.4	381.8	113.3	460.3
Run 6	96.44	366.7	108.1	350.4	92.05	233.9	111.3	347.3
Run 7	109.0	394.1	81.54	336.9	134.3	375.4	149.6	357.9
Run 8	92.39	342.7	70.37	317.5	121.4	387.4	137.3	488.3
Run 9	66.79	319.1	106.2	392.2	109.2	352.6	122.6	388.7
Run 10	91.99	340.8	106.4	332.3	135.4	442.4	153.4	385.9
Run 11	118.1	317.4	96.15	336.9	153.8	460.8	86.28	312.9
Run 12	92.06	329.3	94.12	339.6	89.62	309.0	98.93	383.7

S-site binders	6P3 (loop closed)		6P3 (loop open)		2B4, A		2B4, B		NHI		FX11	
	W[a]	F[b]	W[a]	F[b]	W[a]	F[b]	W[a]	F[b]	W[a]	F[b]	W[a]	F[b]
Run 1	601.9	815.0	140.3	327.3	708.0	1060	629.3	817.5	485.2	798.9	175.9	491.8
Run 2	576.4	850.5	201.6	433.9	620.4	978.5	705.9	1083	453.5	797.5	197.7	506.7
Run 3	649.4	965.4	111.6	334.3	780.1	1138	815.3	939.1	443.4	809.3	209.3	467.0
Run 4	555.2	831.3	194.2	407.3	706.7	1055	707.5	931.8	404.2	745.3	253.0	449.6
Run 5	610.7	801.3	192.0	465.8	745.4	1102	623.6	793.5	441.6	841.0	229.9	425.1
Run 6	594.5	921.1	196.5	382.4	639.3	936.7	872.7	1104	398.3	787.8	186.5	409.2
Run 7	628.8	988.1	172.7	411.3	628.7	981.0	656.1	887.1	447.6	702.4	183.0	418.9
Run 8	612.4	834.3	131.4	305.9	695.2	1039	672.1	830.4	408.6	803.5	253.4	563.1
Run 9	541.1	753.9	175.1	403.2	699.8	1082	630.5	962.9	516.9	803.7	219.3	453.2
Run 10	539.8	763.1	174.9	380.0	674.2	1032	607.3	852.3	437.4	765.1	182.8	380.0
Run 11	542.7	853.1	173.9	412.4	688.6	990.8	670.1	860.4	366.0	712.1	196.5	426.9
Run 12	443.5	691.2	164.3	436.6	557.9	919.1	540.3	774.9	442.6	763.9	201.3	459.4

Dual-site binders	0SN		1E4	
	W[a]	F[b]	W[a]	F[b]
Run 1	795.7	858.7	674.4	648.6
Run 2	681.2	834.9	537.0	585.5
Run 3	829.0	1016	575.0	609.3
Run 4	735.4	824.6	619.4	581.1
Run 5	796.9	892.1	707.6	685.4
Run 6	792.6	875.8	649.0	646.1
Run 7	916.4	931.8	598.6	556.0
Run 8	934.0	985.9	649.3	748.2
Run 9	860.2	916.6	520.4	543.7
Run 10	798.2	830.3	574.1	611.3
Run 11	821.4	889.3	631.5	674.6
Run 12	712.9	802.6	625.1	612.7

[a]Work, with the unit being kJ mol^{-1}.
[b]Peak force, with the unit being kJ mol^{-1} nm^{-1}.
