# Supplementary material for: Human Lactate Dehydrogenase A Inhibitors: A Molecular Dynamics Investigation
Source: PLoS One. 2014 Jan 17;9(1):e86365. doi: 10.1371/journal.pone.0086365 (PMC3895040; doi:10.1371/journal.pone.0086365)
Supplement: Text S7 — Loop conformations for the pulling of S-site inhibitors. (PDF) [file pone.0086365.s011.pdf]

Text S7. Loop conformations for the pulling of S-site inhibitors.

Protein backbones are shown in cartoon while S-site inhibitors are represented by thick sticks.

Closed (2B4 A vs 6P3, loop closed vs NHI$_S$)

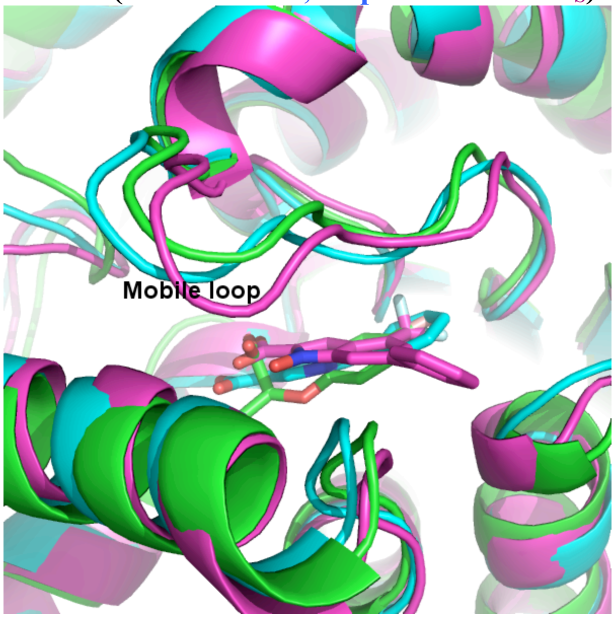

Open (6P3, loop open vs FX11$_S$)

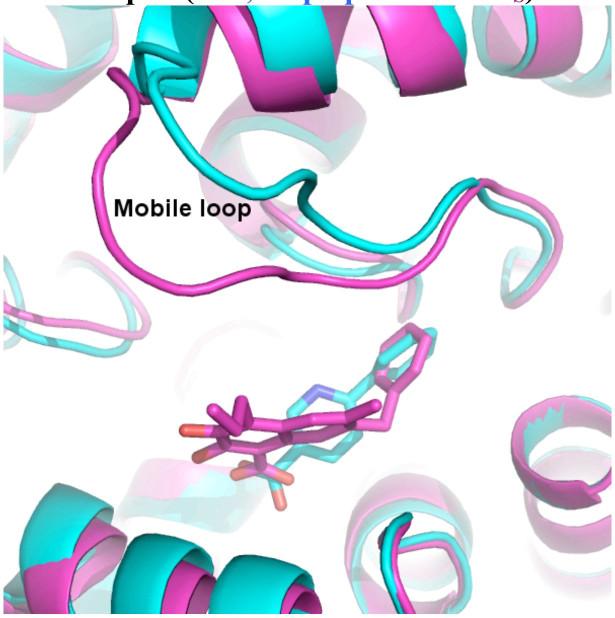

The closed loop conformations are similar among S-site inhibitors 2B4, 6P3, and NHI$_S$. But the open loop conformations are very different between 6P3 and FX11$_S$, which renders their steered MD results incomparable.
